# Accuracy of Non‐Invasive Imaging Techniques for the Diagnosis of MASH in Patients With MASLD: A Systematic Review

**DOI:** 10.1111/liv.16127

**Published:** 2024-10-14

**Authors:** Jennifer Cathcart, Rachael Barrett, James S. Bowness, Ashis Mukhopadhya, Ruairi Lynch, John F. Dillon

**Affiliations:** ^1^ Division of Molecular and Clinical Medicine University of Dundee Dundee UK; ^2^ Gastroenterology Department Aberdeen Royal Infirmary Aberdeen UK; ^3^ University College London Hospitals NHS Foundation Trust London UK; ^4^ Department of Targeting Intervention University College London London UK

**Keywords:** biopsy, imaging, MASH, MASLD, non‐invasive

## Abstract

**Background and Aims:**

Metabolic dysfunction‐associated steatotic liver disease (MASLD) is a growing public health problem. The secondary stage in MASLD is steatohepatitis (MASH), the co‐existence of steatosis and inflammation, a leading cause of progression to fibrosis and mortality. MASH resolution alone improves survival. Currently, MASH diagnosis is via liver biopsy. This study sought to evaluate the accuracy of imaging‐based tests for MASH diagnosis, which offer a non‐invasive method of diagnosis.

**Methods:**

Eight academic literature databases were searched and references of previous systematic reviews and included papers were checked for additional papers. Liver biopsy was used for reference standard.

**Results:**

We report on 69 imaging‐based studies. There were 31 studies on MRI, 27 on ultrasound, five on CT, 13 on transient elastography, eight on controlled attenuation parameter (CAP) and two on scintigraphy. The pathological definition of MASH was inconsistent, making it difficult to compare studies. 55/69 studies (79.71%) were deemed high‐risk of bias as they had no preset thresholds and no validation. The two largest groups of imaging papers were on MRI and ultrasound. AUROCs were up to 0.93 for MRE, 0.90 for MRI, 1.0 for magnetic resonance spectroscopy (MRS) and 0.94 for ultrasound‐based studies.

**Conclusions:**

Our study found that the most promising imaging tools are MRI techniques or ultrasound‐based scores and confirmed there is potential to utilise these for MASH diagnosis. However, many publications are single studies without independent prospective validation. Without this, there is no clear imaging tool or score currently available that is reliably tested to diagnose MASH.

**Summary**

- There is no clear imaging tool or score currently available to diagnose MASH.
- The most promising imaging tools are MRI techniques or ultrasound‐based scores.
- More independent validation studies are needed; this will reduce bias.
- Future work should build on these studies with validation.

**Abbreviations**
AC: attenuation coefficient
ARFI: acoustic radiation force impulse
cT1: iron‐corrected T1 mapping
DS: dispersion slope
GA: gadoxetic acid‐enhanced
MRE: magnetic resonance elastography
MRI‐PDFF: MRI‐derived proton density fat fraction
MRS: magnetic resonance spectroscopy
SPIO: superparamagnetic iron oxide particle
SW: shearwave elastography

## 1 Introduction

The definitions and terminology of fatty liver diseases have recently been revised by a joint consensus of leading experts, endorsed by the American association for study of liver diseases (AASLD) and the European association for study of the liver (EASL). To a spectrum of steatotic liver diseases, of which the disease formally called non‐alcoholic fatty liver disease (NAFLD) would now most closely approximate metabolic dysfunction‐associated steatotic liver disease (MASLD) [1]. MASLD incorporates metabolic dysfunction, the key driver in its pathophysiology. MASLD is estimated to affect 30% of the global population, but with the rise in obesity rates, it is set to increase [2]. Research predicts a 56% rise over the next decade [3]. Thus, it is a public health crisis. Global trends over the past decade have shown a yearly increase in liver transplants for MASLD [4]. In the USA, MASLD is demonstrated to be the fastest growing liver disease over the past five years [5]. It is estimated that MASLD will cost approximately €35 billion annually (ranging between €345 and €1163 per patient) across the UK, Germany, France and Italy and $103 billion ($1613 per patient) in the USA [6]. A German study reported that, in those with advanced disease, mean annual costs were substantially higher, with decompensated cirrhosis costing €22 561, liver transplant patients costing €34 089 and hepatocellular carcinoma (HCC) patients costing €35 910 per patient per year [7]. Dulai et al. found that the stage of liver fibrosis was the strongest predictor for liver‐related mortality and the development of other comorbidities [8].

Metabolic dysfunction‐associated steatohepatitis (MASH, previously known as NASH), the co‐existence of steatosis and inflammation, is a leading cause of progression to fibrosis. The first drug, Rezdiffra (resmetirom), for the treatment of MASH was approved by the FDA in March 2024 [9]. Otherwise, the key management is weight loss strategies. Vilar‐Gomez et al. found the highest rates of NASH resolution and fibrosis regression in patients with weight losses ≥ 10% [10]. Adherence to lifestyle modification to achieve this level of weight loss can be difficult for patients. The BRAVES study showed better resolution of NASH in patients after bariatric‐metabolic surgery versus lifestyle modification alone [11]. Regression of stage F1 fibrosis is more likely than advanced stages with simple lifestyle changes and treatment of the metabolic comorbidities [12]. Therefore, it is important to correctly diagnose the stage of liver fibrosis and to diagnose at an earlier stage. Thus, diagnosis of MASH, the precursor to fibrosis, is the ideal strategy in the management of MASLD. Lassaily et al. showed that MASH resolution alone improves survival, and 95% fibrosis regression occurs in the presence of MASH resolution [13]. For this reason, we wish to focus on the diagnosis of MASH regardless of fibrosis staging. Currently, the gold standard for diagnosis of MASH is via liver biopsy, which carries significant risks, including mortality [14]. Therefore, a better way to diagnose MASH is needed. There are currently multiple biomarkers that have been developed in response to the need to diagnose MASH, but none have gone on to enter clinical practice.

Previous systematic reviews have focused on MASLD or fibrosis but few on MASH diagnosis. Within these reviews, fewer still have focused on imaging and MASH, with the majority covering biomarkers or clinical scores. We found one previous systematic review focusing on steatohepatitis and imaging by Besutti et al. [15]. This is now6 years old, and an updated review would be desirable to incorporate further developments in the field. Therefore, we sought to provide a well‐informed systematic review to add to the sparse literature on the topic. As there is rising pressure for a non‐invasive test for MASH, we felt this review was timely.

## 2 Materials and Methods

The study was performed after reviewing the SIGN Checklist on systematic reviews [16] and the Cochrane handbook of diagnostic test accuracy methodology [17]. It also followed the PRISMA checklist for diagnostic test accuracy [18] and uploaded a study protocol to Prospero: crd.york.ac.uk/PROSPERO/display_record.php?RecordID=465257.

### 2.1 Inclusion Criteria

- Patients with a diagnosis of NAFLD/MASLD and a cohort of patients with a diagnosis of MASH/NASH via biopsy.
- Measurable accuracy outcomes: sensitivity, specificity and/or area under the curve of the receiver operating characteristic (AUROC) with comparison to liver biopsy.

### 2.2 Exclusion Criteria

- Studies on animals or children (Age < 16)
- Studies focusing on fibrosis or steatosis solely
- Abstracts only
- Language other than English

### 2.3 Reference

Studies reporting liver histology showing MASH via liver biopsy.

### 2.4 Main Outcome(s)

Primary outcome – Diagnostic accuracy of the test; sensitivity, specificity and/or AUROC or C‐statistic.

Secondary outcome – Compare the investigation to the component of the NAS (NAFLD Activity Score) if described.

### 2.5 Searches

Eight academic databases were searched in Oct 2023 and updated in Nov 2023: Embase, PubMed, Cochrane, CINAHL, ACM digital, clinicaltrials.gov, WHO ICTRP Search Portal and Open Grey. Advice from an academic librarian was sought to develop and execute a search strategy using a combination of controlled vocabulary and search terms, which was adjusted per database (Supporting Information). Two reviewers (JC and RB) screened the title and abstracts of papers independently after removing duplications using Rayyan Software. Any disagreements were settled between reviewers or adjudicated by a third reviewer (JD). There were no limitations to the publication date. Additionally, references of previous systematic reviews and included papers were checked for additional papers. We also searched trial registries and reported ongoing studies.

### 2.6 Data Extraction and Risk of Bias and Certainty of Evidence Assessment

One reviewer (JC) extracted demographics from the studies using a predesigned check list. This was cross‐checked by a second reviewer (RB). JC and RB evaluated the quality of studies included using the Cochranes quality assessment of diagnostic accuracy studies (QUADAS‐C) tool. Any disagreement between the reviewers over the risk of bias was resolved with discussion between reviewers. JC and RB also used the revised Quality Assessment of Diagnostic Accuracy Studies (QUADAS‐2) evaluation to assess the quality of the papers. JC graded the certainty of evidence using the GRADE (Grading of recommendations assessment, development and evaluation) approach.

## 3 Results

### 3.1 Characteristics of Included Studies

A total of 3647 studies plus seven from other sources were found. This is illustrated in the PRISMA flowchart (Supporting Information). After full text review, this was limited to 83 studies. Within the 83 studies, 30 studies reported results either additionally or solely for at‐risk MASH (NAS ≥ 4 + ≥ F2). Sixteen of these studies were reporting on the FibroScan‐AST (FAST) score alone. Due to a recent systematic review and meta‐analysis on the FAST score in at‐risk MASH [19] and as we wish to focus on MASH diagnosis, regardless of fibrosis staging, we have included results for studies that exclusively report on at‐risk MASH in the Supporting Information, and here focus analysis on the remaining 69 studies that include results for MASH diagnosis regardless of fibrosis staging, published from 2002 to 2023 [20, 21, 22, 23, 24, 25, 26, 27, 28, 29, 30, 31, 32, 33, 34, 35, 36, 37, 38, 39, 40, 41, 42, 43, 44, 45, 46, 47, 48, 49, 50, 51, 52, 53, 54, 55, 56, 57, 58, 59, 60, 61, 62, 63, 64, 65, 66, 67, 68, 69, 70, 71, 72, 73, 74, 75, 76, 77, 78, 79, 80, 81, 82, 83, 84, 85, 86, 87, 88]. The two largest groups of imaging papers were on MRI and ultrasound. There were 31 studies on MRI, 27 on ultrasound, five on CT, 12 on transient elastography, eight on controlled attenuation parameter (CAP) and 2 on scintigraphy. Additionally, there were eight clinical trials. Several studies looked at more than one imaging technique. Table 1 summarises the principles of the different imaging modalities and the theory for MASH diagnosis.

**TABLE 1:** Summary of underlying principles of imaging methods and theory in diagnosing MASH.

	Underlying principles	Theory in diagnosing MASH
*MRI*		
MRE	Generates mechanical shear waves by passing low‐frequency vibrations through the liver delivered via a pad placed over the patient's liver. The wavelength reflects stiffness of the tissue	Detects increased stiffness of liver secondary to developing fibrosis and inflammation related to MASH
MRI‐PDFF	Compares the fat and water components of an MRI scan, and expresses as a percentage	Detects increased steatosis, which could be a marker of increased risk of MASH. Positive correlations between change in MRI‐PDFF and steatosis grade have been demonstrated [40, 77]
MpMRI	Combines different MRI techniques into a single scan. T1 mapping detects increases in extracellular fluid. Hepatic iron can be a confounding factor. Therefore, iron is calculated with parallel acquisition of T2. Iron‐corrected T1 relaxation maps (cT1) take out this confounding factor	cT1 or T1 mapping correlates with fibrosis and inflammation. Inflammation creates increases in extracellular fluid detected by the scan [64] Liver*MultiScan Software (*Perspectum, Oxford, UK) postprocesses non‐contrast MpMRI imaging scans to simultaneously quantify steatosis (via MRI‐PDFF), fibrosis and inflammation (via cT1)
GA‐MRI	Gadoxetic acid is a hepatobiliary contrast agent that is taken up by the hepatocytes, allowing more detailed interpretation of liver anatomy	The hepatobiliary phase T1 relaxation time potentially differentiates MASH from steatosis. Amorim et al. hypothesise that expression of the multidrug resistance protein transporter is increased in patients with MASH, causing an increase in GA excretion with a consequent decrease in signal intensity [24]. Bastati et al. hypothesise the reduction in signal intensity is due to liver injuries reducing hepatosinusoidal space and thus GA uptake [26]
MRI	MRI creates a magnetic field, which forces protons in the body to align with that field. The protons are then stimulated by a radiofrequency current. When the radiofrequency field is turned off, the MRI sensors detect the energy released as the protons realign with the magnetic field. The difference between various types of tissues is based on these magnetic properties	Different methods Gallego‐Duran et al.: Utilise three MRI variables from three different MRI protocols and call then NASHMRI; they found them to be independently associated with MASH [36] Leporq et al.: Magnetic susceptibility; the magnetic susceptibility decreased in MASH. Felt to be related to diamagnetic material changes in fibrosis and inflammation [55]
IVIM	Based on the random movement of individual water protons, which results in molecular diffusion, called Brownian motion. The mean square distance travelled by a molecule is proportional to time and diffusion coefficient (D). The scan also measures the perfusion component (D*) and the vascular volume fraction (f)	Accumulation of fat, inflammation and hepatocellular ballooning, present in MASH, reduce sinusoidal space and cause restrictive barriers detected as reduced diffusion (D). D* and f indicate change that occurs in the liver blood flow because of distortion of the microcirculatory anatomy and the compression of the sinusoidal space related to inflammation. Additionally, fibrosis can also influence D, D* and f [83]
MRS	The distribution of electrons within an atom causes nuclei within different molecules to have a slightly different magnetic field. This results in slightly different resonant frequencies, which creates an image from the different signals. This allows the presence and concentration of various metabolites to be determined from tissues	Inflammation related to MASH causes different metabolic changes, which can be detected by MRS. Kim et al. use ^1^H‐MRS (proton MRS) with long echo time; among other metabolites, they looked at Ala peaks, which are shown in other studies to be specific biomarkers for liver injury and indicative of liver enzymes raised by inflammation [48]. Abrigo et al. used phosphorus MRS (^31^P‐MRS) and found a decreased alpha nucleotide triphosphate (α‐NTP)/total phosphate (TP) (*p* < 0.001) ratio in patients with MASH [20]
SPIO	SPIO nanoparticles are a type of contrast agent used in MRI. It enhances MRI by shortening the T1 and T2/T2* relaxation times	Hepatic Kupffer cells effectively remove SPIO from the circulating blood via phagocytosis; thus, they can be imaged using hepatic signal intensity changes. As Kupffer cells are felt to be involved in the pathogenesis of MASH, the changes could be diagnostic of MASH [73]
*Ultrasound*		
Ultrasound scores	A transducer transmits sound waves through the body. The sound waves are reflected to the transducer as echoes. The scanner then calculates the distance from the transducer to the tissue boundary and generates images of tissues based on the distance	There are several different specific parameters seen on ultrasound which have been combined in the studies to create scores that diagnose MASH. These either focus on parameters due to increased steatosis, for example, echogenicity of the liver and hepatorenal angle, which have been found to increase the likelihood of MASH. Or parameters affected by inflammation or steatosis, such as blurring of specific anatomy such as vessels, diaphragm and gallbladder [21, 25, 57]. Ultrasound can also utilise 2D SWE and other techniques as below
RF	RF data alters are related to microstructure changes within tissues [37]	Mechanisms are unclear; however, Gao et al. hypothesise that the selected parameters used in their study reflect the intensity and scattering of liver ultrasound signals and are related to the microstructural changes due to liver inflammation [37]
Microbubbles	2D ultrasound with microbubble contrast agent, which is phagocytosed by liver Kupffer cells, enhancing the liver parenchyma	Contrast enhancement is decreased in patients with MASH, compared to NAFLD patients. This is felt to be explained by the changes in the sinusoidal endothelial system [41]
ARFI	A brief acoustic radiation force impulse is passed through tissue to create displacement. It detects liver stiffness	Detects increased stiffness of the liver secondary to developing fibrosis and inflammation
TE	Mechanical vibration is applied over the liver to create shearwaves. The wavelength is reflective of liver stiffness	Detects increased stiffness of the liver secondary to developing fibrosis and inflammation
SWE	It can be 2D ultrasound shear wave or point shear wave elastography. A transducer induces an elastic shear wave in the underlying tissue. The ultrasound measures the velocity of the shear wave through the tissue. In pSWE, the measurement of stiffness is conducted by targeting a single point in a localised region of interest. This is different from 2D‐SWE, in which the device calculates the mean stiffness within a larger area	SWE can detect changes in stiffness of liver tissue, which reflect increases in inflammation and fibrosis. This stiffness can be measured via three different methods Liver stiffness in kilopascals (kPa) Dispersion slope in metres/second/kilohertz ((m/s)/kHz): parameter used to measure tissue viscosity by measuring the change in shear wave speed with frequency Speed in metre per second (m/s): higher shear wave speeds are associated with higher liver stiffness
CAP	Measures the attenuation of an ultrasound beam as it passes through the liver	Fat affects ultrasound propagation; thus, attenuation is correlated to the percentage of steatosis. Increased steatosis could be a marker of increased risk of MASH
*Other*		
CT	A narrow beam of X‐rays is aimed at a patient and rotated around the body; this produces signals that are processed by a computer to generate cross‐sectional images. The number of X‐rays that pass through the object is inversely proportional to the density of the object	Several different methods Dichtel et al. and Naganawa et al. describe using different CT texture analysis methods for diagnosis [29, 61]. Alluding to a change of the texture of the liver in MASH Kim et al. describe using the liver‐spleen Hounsfield unit (HU), which is a tool to look at hepatic fat content in comparison to spleen. They found it also correlated with the degree of inflammation [46]
Scintigraphy	A radioisotope (^99m^Tc‐phytate), which is a radioactive compound that emits gamma rays, is administered; this is then detected by a gamma camera. This creates images showing the distribution of the radioisotope within the body	The reduction of this radioisotope uptake during the scintigraphic exam could be a marker of MASH as ^99m^Tc‐phytate accumulates in the liver, followed by phagocytosis by Kupffer cells. The reticuloendothelial dysfunction of Kupffer cells is involved in the pathogenesis of steatohepatitis [67]

Abbreviations: ARFI, acoustic radiation force impulse; CAP, controlled attenuation parameter; CT, computerised tomography; GA MRI, Gadoxetic acid–enhanced MR; IVIM MRI, intravoxel incoherent motion; MpMRI, multiparametric magnetic resonance; MRE, magnetic resonance elastography; MRI‐PDFF, MRI proton density fat fraction; MRS, magnetic resonance spectroscopy MRI; SPIO, Superparamagnetic iron oxide; SWE, shear wave elastography; TE, transient elastography.

### 3.2 Demographics

We report data on subject age, gender, body mass index (BMI), race, country of origin and year located in the Supporting Information.

### 3.3 Histopathological Analysis

Most (30/69 studies) biopsies were percutaneous via ultrasound guidance; the reminder took place intra‐operatively, or included both methods, or did not report on the type of biopsy. The pathological definitions of MASH were heterogeneous. The criteria used to diagnose included Brunt criteria, NAS, NASH clinical research network in non‐alcoholic hepatitis (NASH CRN), fatty liver inhibition of progression (FLIP) algorithm, steatosis, activity and fibrosis (SAF) score and the Matteoni classification. The most common score was the NAS score, which is calculated by adding scores of three principle components: steatosis (0–3), lobular inflammation (0–3) and hepatocyte ballooning (0–2). The NAS score makes up the NASH CRN score, but NASH CRN also has a separate scoring system for fibrosis, which can be added. The Brunt criteria includes these three parameters but additionally reports on fibrosis. Brunt defines NASH by the presence of fibrosis (≥ grade 1) or necroinflammation (≥ grade 2). It also groups inflammation and ballooning together [89]. The SAF score comprises the grade of activity from A0 to A4, grading both ballooning and lobular inflammation together, and the stage of fibrosis from F0 to F4. The fibrosis staging is like the NASH CRN criteria except it pools the 3 substages (1 a, 1b and 1c) into a single F1 score. Compared with NAS, the SAF activity score equally weights ballooning and lobular inflammation. SAF > 1 is considered mild disease, > 2 moderate disease and > 3 severe disease. The FLIP algorithm diagnoses NASH when at least one of each of the three principle features is present. This contrasts to the NAS score, which can diagnose NASH without one of the components being present [90]. The Matteoni classification defines NAFLD into four types. Types 3 and 4 are what are used in the papers to describe NASH. Type 3; steatosis and hepatocyte ballooning, or type 4; type 3 and Mallory's hyaline or fibrosis [71]. Even within papers using the NAS score, different cut‐offs were used for the definition of MASH from NAS ≥ 3 to NAS ≥ 5. Several studies also reported using NASH CRN criteria but did not report the specific NAS score used. Other studies had individual definitions of MASH based on the three principle components. Wildman‐Tobriner et al.'s study combined 3 trial cohorts, in which two of the cohorts needed fibrosis stages 1–3 to be enrolled in the study [77].

### 3.4 Risk of Bias

We evaluated the papers using the Cochranes QUADRAS‐C toolkit (see Supporting Information). The risk of bias was widespread in the included papers, but no study scored high risk for all four categories: patient selection, index test, reference standard and flow and timing. However, nine studies scored either unclear or high risk within all four categories [22, 32, 41, 44, 65, 77, 83, 85, 86], 11 studies were at risk of selection bias as they only used patients undergoing bariatric surgery [21, 22, 30, 32, 38, 39, 49, 57, 65, 67, 86] and one study excluded all those with BMI > 40 or seen in the obesity clinic [72]. One study only included patients with type 2 diabetes [83], one study excluded all patients with type 2 diabetes on insulin [25] and a further study excluded those on glucose‐lowering tablets [80]. Three studies included clinical trial patients [40, 56, 77]; one of these excluded any patient with uncontrolled diabetes [77]. Whilst all studies included patients with liver biopsies, ten had a control group that did not undergo a liver biopsy [20, 27, 31, 33, 41, 44, 48, 68, 72, 85]. However, only three studies included the non‐biopsied control group in their diagnostic accuracy results [20, 41, 44]. For the paper by Lirussi et al., it was unclear if they included the control group in their results [85]. One study, Zhou et al. included 23 biopsied controls [81]. Only 14/69 studies had pre‐set thresholds for their imaging test or undertook a validation [34, 36, 37, 42, 53, 59, 60, 61, 69, 70, 75, 81, 86, 88]. Macias et al. validated their study but only gave negative predictive value (NPV) and positive predictive value (PPV) from the study [59]. The remainder (55/69 studies) were regarded as high risk of bias for the index test category within the tool. In all, 36 studies were scored as either unclear or high risk of bias after excluding patients from analysis due to missing data, or unreliable/unachievable scan results or inadequate liver biopsies [20, 21, 22, 23, 24, 26, 29, 31, 32, 33, 34, 35, 37, 38, 41, 42, 43, 44, 47, 49, 50, 51, 52, 54, 58, 59, 60, 62, 63, 64, 65, 71, 77, 83, 86, 88].

### 3.5 Certainty of Evidence

The level of certainty of evidence, according to GRADE criteria, is reported for the different imaging methods in the Supporting Information.

### 3.6 Data Synthesis

We grouped studies into similar imaging methods to compare. Where there was a testing and validation cohort, we reported the outcomes of the validation cohort. Results are presented in tables to the nearest two decimal points. We undertook a narrative synthesis of the data. Due to the heterogeneity of the data, different variations on imaging tests, different MASH definitions and different thresholds for tests, data pooling was not possible.

### 3.7 MASH Diagnosis

#### 3.7.1 Magnetic Resonance Imaging (MRI)

There were 31 studies reviewing MRI and steatohepatitis presented in Table 2.

**TABLE 2:** Diagnostic accuracy of MRI studies in MASH diagnosis.

Method/model used	Authors and year	AUROC (95% CI)	Population number (MASH)	Pathology definition	Sensitivity and specificity
*MRE or MRE with other techniques*					
2D MRE	Allen et al. 2020 [22] Alsaqal et al. 2021 [23] Chen et al. 2011 [28] Costa‐silva et al. 2018 [27] Lee et al. 2020 [54] Park et al. 2017 [63] Troelstra et al. 2021 [75, 76] Loomba et al. 2016 [87] Imajo et al. 2021 [43] Li et al. 2023 [56] Loomba et al. 2014 [88]	0.61 (CI 0.53–0.69) cross‐validated C‐statistic for NASH diagnosis and NAS prediction 0.64 (CI 0.58–0.70). Both combined cohort 0.74 (CL N/A) 0.93 (CI N/A) 0.79 (0.66–0.95) 0.77 (CI 0.69–0.84) 0.70 (CI 0.57–0.82) 0.79 (CI N/A) MRE G1 + MRE G2 0.69 (CI N/A) 0.75 (CI N/A) 0.57 (CI 0.47–0.67) 0.73 (CI N/A) 0.73 (CI N/A)	175 (81) 68 (53) 58 (36) 49 (25) 130 (70) 104 (72) 37 (22) 100 (72) 145 (87) 104 (45) 117 (106)	The presence of steatosis with (1) ballooning or (2) lobular inflammation with associated fibrosis SAF grading Brunt criteria NAS ≥ 5 > 5% steatosis and inflammation with hepatocyte ballooning NAS ≥ 3 NAS ≥ 3. Steatosis and ballooning grade and lobular inflammation grade ≥ 1 for each NAS ≥ 5 NASH was defined as NAS ≥ 4 and at least ballooning ≥ 1 and inflammation ≥ 1 NASH CRN; no specific NAS NAS ≥ 3	N/A 0.49 se and 1.00 spec cut‐off > 2.74 kPa 0.94 se and 0.73 spec cut‐off > 2.74 kPa and 0.83 se and 0.82 spec cut‐off > 2.90 kPa 0.72 (0.48–0.86) se and 0.88 (0.66–0.97) at cut‐off > 3.24 kPa N/A 0.64 se and 0.68 spec for cut‐off 2.53 kPa 0.70 se and 0.87 spec for cut‐off 2.27 kPa MRE G1 + 0.45 se and 1.00 spec for 0.88 kPa cut‐off MRE G2 N/A N/A N/A 0.43 se and 0.89 spec (cross‐validated) at 3.26 kPa
3D MRE	Loomba et al. 2016 [87]	0.76 (60 Hz) (CI N/A) 0.74 (40 Hz) (CI N/A)	100 (72)	NAS ≥ 5	N/A
3D‐MRE + MRI‐PDFF	Allen et al. 2020 [22]	0.73 (CI 0.65–0.81) cross‐validated C‐statistic for NASH diagnosis and 0.82 (CI 0.77–0.87) for predicting the NAS score	175 (81)	The presence of steatosis with (1) hepatocellular ballooning or (2) lobular inflammation with associated fibrosis	0.67 se and 0.80 spec for 3D MRE model
2D MRE + MRI‐PDFF	Alsaqal et al. 2021 [23] Li et al. 2023 [56]	0.84 (CI N/A) 0.79 (CI N/A)	68 (53) 104 (45)	SAF grading NASH CRN. No specific number	0.74 se and 0.87 spec cut‐off > 1.38 kPa N/A
Machine learning model with MRE + MRI‐PDFF	Dzyubak et al. 2021 [30]	0.87 (CI N/A) for NASH diagnosis + 0.85 (CI N/A) cross‐validated C‐statistic of for both manual and automated measurements for model to predict NAS score	83 (34)	NAS score, does not specify a number	N/A
MRE‐based NASH score with demographic and laboratory variables	Lee et al. 2022 [53]	0.84 (CI 0.77–0.91) + C‐statistic 0.81 at bootstrapping	127 (69)	Steatosis was ≥ 5% with inflammation and ballooning of hepatocytes	0.68 se and 0.91 spec for cut‐off 0.68 0.91 se and 0.55 spec for cut‐off 0.37
MRE + T1 mapping	Li et al. 2023 [56]	0.74 (CI N/A)	104 (45)	NASH CRN. No specific number	N/A
MRI‐PDFF + T1 + MRE	Li et al. 2023 [56]	0.83 (CI 0.75–0.92)	104 (45)	NASH CRN. No specific number	N/A
*Non‐MRE‐based MRI scores*					
MRI‐PDFF	Alsaqal et al. 2021 [23] Troelstra et al. 2021 [76] Imajo et al. 2021 [43] Garteiser et al. 2021 [38] Idilman et al. 2021 [40] Li et al. 2023 [56] Wildman‐Tobriner et al. 2018 [77]	0.70 (CI N/A) NASH 0.57 (CI N/A) PDFF MRI‐M + PDFF MRS 0.56 (CI N/A) + PDFF Dixon 0.52 (CI N/A) 0.80 (CI: 0.73–0.87) 0.84 (CI 0.77–0.91) 0.64 (CI 0.54–0.73) 0.82 PDFF Dixon (CI 0.72–0.91) 0.72 (CI 0.59–0.84)	68 (53) 37 (22) 145 (87) 152 (30) 135 (73) 104 (45) 170 (51)	SAF Steatosis and ballooning grade was ≥ 1 and lobular inflammation grade ≥ 1 was present NAS ≥ 4 and at least one in ballooning ≥ 1 and inflammation ≥ 1 FLIP criteria NASH CRN. No specific number NASH CRN. No specific number NAS ≥ 4. Combined trial cohorts of 3 trails but uses only two trails for NASH diagnosis. One trial NAS ≥ 4 and ≥ 1 hepatocyte ballooning and other trial NAS ≥ 4 with ≥ 1 hepatocyte ballooning, inflammation and steatosis. Fibrosis stage 1–3 was needed for enrolment	0.81 se and 0.60 spec 0.41 se and 0.87 spec for 23.72% cut‐off + 0.46 se and 0.73 spec for 21.19% cut‐off + 0.33 se and 0.87 spec for 21.84% cut‐ off N/A 0.97 (CI 0.86–1.00) se and 0.60 (CI 0.50–0.71) spec at 12% cut‐off 0 0.61 (0.49–0.72) se and 0.65 (0.51–0.76) spec N/A 0.75 (CI 0.35–0.97) se and 0.75 (CI 0.67–0.81) spec cut‐off 12.4%
T1 mapping and MRI PDFF	Imajo et al. 2021 [43] Li et al. 2023 [56]	0.83 (CI: 0.76–0.90) 0.81 (CI N/A)	145 (87) 104 (45)	NAS ≥ 4 and at least one in ballooning ≥ 1 and inflammation ≥ 1 NASH CRN. No specific number	N/A N/A
T1 mapping	Eddowes et al. 2018 [31] Li et al. 2023 [56]	0.69 (0.50–0.88) Simple steatosis and NASH 0.74 (0.59–0.88) NAS < 5 versus NAS ≥ 5 0.72 (CI N/A)	50 (38) 104 (45)	Presence of lobular inflammation and hepatocyte ballooning. Secondly, NASH CRN score was used for grading NASH CRN. No specific number	N/A
M‐PAST – MRI‐PDFF and AST	Imajo et al. 2023 [42]	0.81 (CI 0.73–0.86) validation cohort In the UCSD cohort (non‐Japanese cohort), M‐PAST (0.77) (CI 0.71–0.83)	169 (98) validation cohort 234 (117) USCD cohort	NAS ≥ 4	N/A
IVIM	Troelstra et al. 2021 [76] Parente et al. 2015 [83]	0.73 (CI N/A) IVIM D + IVIM D* 0.58 (CI N/A) + IVIM f 0.68 (CI N/A) 0.74 (CI N/A) D + D* 0.69 (CI N/A) + F 0.61 (CI N/A)	37 (22) 59 (22)	Steatosis and ballooning grade was ≥ 1 and lobular inflammation grade ≥ 1 was present Steatosis and mixed‐cell inflammatory infiltrates in the hepatic lobules and damage of hepatocytes. Damage characterised by contiguous patch of hepatocytes showing prominent ballooning with or without apoptosis and necrosis	0.86 se and 0.53 spec for 0.0012 mm^2^/s cut‐off + 0.81 se and 0.53 spec for 22.90% cut‐off + 0.48 se and 0.80 spec for 0.10 mm2/s cut‐off D 0.69 se and 0.66 spec cut‐off 0.76 D* 0.69 se and 0.71 spec cut‐off 0.42 F 0.49 se and 0.70 spec cut‐off 0.34
NASH MRI – model based on independent MRI variables	Gallego‐Duran et al. 2016 [36]	0.83 (CI 0.73–0.93) Validation cohort	87 (44) Validation cohort	Patterns of histological distribution focusing on inflammation and ballooning	0.87 se and spec 0.60 cut‐off 0.5
Magnetic susceptibility	Leporq et al. 2017 [55]	0.91 (CI 0.79–1.0)	32 (20) Validation cohort	FLIP criteria	N/A
MR – MASH score – MRI PDFF and waist circumference (in MR images) and height	Marti‐Aguado et al. 2023 [60]	0.86 (CI 0.77–0.92) Validation cohort	63 (59) validation cohort	NAS ≥ 4 presence of steatosis, lobular inflammation and ballooning with or without fibrosis	0.91 se and 0.73 spec (rule out MASH) (MR–MASH score < 400) 0.56 se and 0.87 spec (rule in MASH) (MR–MASH score ≥ 600)
LiverMultiScan measuring LIF scores from T_1_ and T_2_	Pavlides et al. 2017 [64]	0.80 (CI 0.69–0.92)	71 (46)	FLIP criteria	0.91 (0.79–0.98) se and 0.52 (0.31–0.72) spec 1.4 LIF cut‐off
MRI parameters	Saadeh et al. 2002 [70]	None of the radiological features included could distinguish patients with the pathologic diagnosis of NASH from steatosis	25 (17)	Type 3 (steatosis, lobular inflammation, hepatocyte ballooning) or type 4 (steatosis, lobular inflammation, hepatocyte ballooning and Mallory's hyaline or fibrosis)	N/A
Multiparametric MR index	Kim et al. 2020 [82]	0.88 (CI N/A)	47 (20)	SAF criteria	0.95 se and 0.78 spec cut‐off of 4.6 + 0.80 se and 0.85 spec cut‐off of 6
GA‐MRI	Amorim et al. 2020 [24] Bastati et al. 2014 [26]	0.68 (CI N/A) 0.85 (0.75–0.91 CI)	56 (38) 81 (35)	NASH CRN, does not specify a NAS score SAF – Activity score > 2 and any fibrosis/steatosis	0.47 se and 0.83 CEI cut‐off 1.76 + 0.92 se and 0.28 spec CEI cut‐off 2.00 + 0.32 se and 0.94 spec CEI cut‐off 1.66 0.97 (CI 84.7–99.9) se and 0.63 (CI 47.5–76.8) spec cut‐off 1.24
MRS	Abrigo et al. 2014 [20] Kim et al. 2017 [48]	0.71 (0.62–0.79 CI) 1.00 (CI 1.00–1.00) for Ala + 0.78 (CI 0.61–0.96) for Lac + TG	151 (91) 32 (11)	Matteoni classification – type 3 and type 4 were defined as NASH NAS ≥ 5	0.91 se and 0.16 spec α‐NTP/TP cut‐off of ≤ 16.36% + 0.28 se and 0.91 spec cut‐off of ≤ 10.57% 1.00 se and 1.00 spec cut‐off value > 16.04% Ala 0.82 se and 0.67 spec at a cut‐off value > 360.8% Lac + TG
SPIO‐enhanced	Tomita et al. 2008 [73] Smits et al. 2016 [68]	0.79 (CI 0.56–1.01) T values + %T2 values 0.83 (CI 0.64–1.02) 0.87 (CI 0.72–1.00)	19 (10) 24 (13)	NAS ≥ 5 NAS ≥ 5 when steatosis, inflammation and ballooning were present	Se 1.0 and 0.67 spec cut‐off tau value of 42.8 + 0.88 se and 0.73 spec cut‐off %T2 value of 32.5 0.77 se and 0.91 spec at ΔR2* < 45.5 s^−1^ cut‐off 0.92 se and 0.55 spec cut‐off < 59.2 s^−1^

Abbreviations: AST, aspartate aminotransferase; CEI, contrast enhancement index; CI, Confidence Interval; FLIP, fatty liver inhibition of progression; GA‐MRI, gadoxetic acid‐enhanced MRI; IVIM D*, pseudodiffusion coefficient; IVIM D, diffusion coefficient; IVIM, intravoxel incoherent motion; kPa, kilopascals; LIF, liver inflammation and fibrosis score; mm^2^/s, millimetres per second; MRE, magnetic resonance elastography; MRE G1, shear modulus; MRE G2, loss modulus; MRS, magnetic resonance spectroscopy; NAS, NAFLD activity score; PDFF Dixon, three‐point DIXON proton density fat fraction; PDFF MRS, magnetic resonance spectroscopy proton density fat fraction; PDFF, proton density fat fraction; PDFF‐M, magnitude‐based MRI proton density fat fraction; SAF, steatosis activity and fibrosis score; Se, sensitivity; Spec, specificity; SPIO, Superparamagnetic iron oxidesg.

##### 3.7.1.1 Non‐MRE Studies

The best AUROC within the MRI studies was 1.0 reported by Kim et al. using magnetic resonance spectroscopy (MRS). The study looked at metabolic changes using ^1^H‐MRS with long echo time [48]. A larger study on MRS was by Abrigo et al., which showed a lower 0.71 AUROC [20]. Both these studies suffered from bias due to not having preset thresholds. However, the Abrigo study was at overall higher risk of bias due to including a non‐biopsied control group in its results as well as missing data due to unsuccessful MRS in 8 patients [20]. Other techniques include gadoxetic acid‐enhanced MRI (GA‐MRI) used by Bastati et al. with an AUROC of 0.85 [26] but this was not replicated in a further study by Amorim et al. (AUROC 0.68) [24]. Apart from having no preset thresholds, both studies were at lower risk of bias. Both had to exclude a low number of patients due to motion artefacts on MRI (3 in Amorim and 10 in Bastati), though Bastati did additionally exclude 7 patients with incomplete biopsy results [24, 26]. MRI‐PDFF scored AUROCs of 0.52–0.84. The highest AUROC was by Garteiser et al.; MRI‐PDFF had 97% sensitivity and 60% specificity, 50% PPV and 98% NPV for diagnosing NASH at a cut‐off of 12% [38]. These results suggest that MRI‐PDFF is better at MASH exclusion rather than diagnosis. This high sensitivity was not reflected in the other studies with lower sensitivities, even in the study by Wildman‐Tobriner et al., who used a similar cut‐off at 12.4%, reporting a sensitivity of 75% [77]. Troelstra et al. conducted PDFF with three different MRI techniques, such as MRS, Dixon and magnitude‐based, but achieved the lowest AUROCs among the studies [76]. MRI‐PDFF scored higher AUROCs when combined with other modality such as MRE in the study by Dzyubak et al. reporting an AUROC of 0.87 for NASH diagnosis and a cross‐validated C‐statistic of 0.85 for NAS prediction with additional use of machine model learning analysis of the imaging data. However, this was in a group of bariatric surgery patients, making this study at risk of selection bias [30]. A few other studies combining 2D MRE and MRI‐PDFF scored lower AUROCs of 0.84 [23] and 0.79 [56], and the 3D MRE and MRI‐PDFF combination resulting in 0.73 [22]. This latter study by Allen *et al*. in a bariatric cohort again shows better results for NASH exclusion than diagnosis [22]. Leporq et al. reported on a decrease in magnetic susceptibility in MASH using MRI; the study achieved an AUROC of 0.91. Apart from no preset thresholds, the paper otherwise scored low risk of bias; however, it was unclear from the paper whether the pathologist was blinded to the results of the MRI [55].

##### 3.7.1.2 MRE Studies

The second best AUROC overall for MRI studies was reported by Chen et al. at 0.93 in MRE. This was using Brunt criteria as the definition of NASH, discriminating patients with NASH from those with simple steatosis in a population of 58 NAFLD patients [28]. Other studies reporting on MRE alone report AUROC ranging from 0.57 to 0.87. The lowest score was a study by Imajo et al. Its pathology definition did not include fibrosis [43]. If we use the cut‐off for definite NASH via the NASH CRN definition (NAS ≥ 5), then the highest AUROC by study is by Costa‐silva et al., who report an AUROC of 0.79 for MRE [27]. The studies by Chen and Costa‐Silva, apart from no preset thresholds, scored otherwise low for bias [27, 28]. The study by Imajo, however, scored high risk of bias due to no preset thresholds, and it was unclear if the pathologist was blinded (although they used 3 different pathologists to score the biopsies). There was a large volume of missing data in this paper, but it only had one missing MRE test [43].

#### 3.7.2 Ultrasound

##### 3.7.2.1 Ultrasound, Shear Wave (SW), Acoustic Radiation Force Impulse (ARFI) and MASH

There are 27 ultrasound‐based studies: five did not report AUROC, including one because it didn't reach statistical significance to build a model (Table 3). Within the studies, the AUROC ranged from 0.65–0.94. The highest AUROC for ultrasound studies was 0.94 jointly by two studies. Firstly, by Funada et al., they developed a model called the NASH pentagon, which included five parameters: SW speed (SWS), SW dispersion slope (SWD), attenuation imaging value (also named attenuation coefficient (AC)), Fib‐4 and BMI [35]. Secondly by Jang et al., who created a risk score based on ultrasound, AC and DS [44]. Funada et al.'s test suffered bias as they had no preset thresholds and not everyone in the study got a biopsy, although they only used biopsied patients in the results [35]. Jang also suffered from no preset thresholds, and it was unclear if the pathologist was blinded. They also included living donors. However, in the Supporting Information, excluding patients for living donation, the AUROC was 0.93 (95% CI: 0.84–0.98) for the risk score [44]. All the studies with SW elastography (SWE) utilised 2D‐SWE rather than point‐SWE (pSWE). The studies with the highest sensitivity (100%) and specificity (100%) were by Lijima et al. They employed the use of a microbubble contrast agent; although the study did show a decrease in uptake of contrast agents for patients with NASH, there was no correlation to the degree of fibrosis or steatosis. The study did not report an AUROC, and we found no other similar study [41].

**TABLE 3:** Diagnostic accuracy of ultrasound, SW and ARFI in MASH diagnosis.

Model used	Authors and year	AUROC (95% CL)	Population number (MASH)	Path grading	Sensitivity and specificity
*Model using 2D US*					
US‐FLI based on liver/kidney contrast, posterior attenuation of ultrasound beam, vessel blurring, gallbladder wall blurring, diaphragm blurring and areas of focal sparing	Ballestri et al. 2012 [25] Nelson et al. 2020 [84] Sourianarayanane et al. 2023 (POCUS) [69]	0.80 (CI 0.676–0.916) 0.93 (CI 0.83–0.97) when the US‐FLI ≥ 5 in normal versus NASH + for steatosis versus NASH was 0.65 (CI 0.56–0.72) when the US‐FLI ≥ 5 0.78 (CI N/A) + When weighted for the effects of BMI, 0.71 (CI N/A)	53 (35) 208 (105) 113 (78) 82 had POCUS scans	NAS > 5 was considered compatible with the diagnosis of severe NASH But ≥ 5 used for AUROC Brunt classification NASH CRN. No specific NAS number given	Se N/A and 0.46 spec at US FLI < 4 0.7 se and 0.93 spec US‐FLI score ≤ 4 + US‐FLI score ≥ 5 0.93 se and 0.57 spec in normal versus NASH + in steatosis versus NASH US‐FLI ≤ 4 Se 0.91 and spec 0.18 + US‐FLI score ≥ 5 Se 0.78 and spec 0.56 0.81 se and 0.63 spec at cut‐off USFLI ≥ 6 + USFLI ≤ 3, 1. 0 ruling out NASH. When weighted for the effects of BMI 0.82 se and spec 0.51
Attenuation coefficient	Jang et al. 2022 [44] Lee et al. 2021 [50] Sugimoto et al. 2020 [71]	0.84 (CI 0.76–0.90) 0.84 (CI 0.75–0.90) 0.71 (CI 0.58–0.83)	132 (53) 102 (55) 111 (90)	NAS > 3 + ≥ 1 point for each category – steatosis, inflammation, ballooning NAS > 3 + ≥ 1 point for each category – steatosis, inflammation, ballooning Matteoni classification – type 3 and type 4 were defined as NASH	0.81 se and 0.80 spec 0.78 se and 0.85 spec N/A
Echogenicity of liver, visualisation of blood vessel, diaphragm and posterior right lobe liver	Manasra et al. 2021 [21]	N/A	45 (19)	≥ 5 NAS with presence of steatosis, inflammation and ballooning. Or NAS 3–4 with grade 1 steatosis and ballooning	1. 0 se and 0.28 spec
Antwerp NASH severity score (ALT, US echogenicity and posterior beam attenuation and C‐peptide)	Francque et al. 2012 [34]	0.88 (CI N/A) Validation cohort	313 (61)	NAS ≥ 5	0.96 se and 0.46 spec
US + ‘NASH pentagon’ (five parameters of 2D SW (speed), SW (DS), attenuation imaging value, Fib‐4 index and BMI	Funada et al. 2023 [35]	0.94 (CI N/A) Biopsied cohort	31 (25) biopsied cohort	Comprehensive diagnosis to include macrovesicular steatosis, ballooning degeneration of hepatocytes, inflammation and apoptotic bodies in the lobules, Mallory‐Denk bodies and fibrosis	N/A
US + Radiofrequency	Gao et al. 2022 [37]	0.82 (CI 0.73–0.91) Validation cohort	259 (100) total cohort and 87 validation cohort	NAS ≥ 5	0.87 se and 0.67 spec at cut‐off QUS ≤ 6.0 0.38 se and 0.90 spec at cut‐off QUS ≥ 7.5
US+ microbubble contrast	Lijima et al. 2007 [41]	N/A	76 (21)	Brunt criteria	1.0 se and 1.0 spec at cut–off 43.6 signal intensity at 20 min 1.0 se and 0.95 spec at cut‐off 137.8 Signal intensity at 5 min
US PATT	Lirussi et al. 2009 [85]	0.75 (CI N/A)	108 (27)	NAS ≥ 3 borderline NASH	1.0 se and 0.5 spec cut‐off value of PATT of 11.8 mm
US + AC and DS risk score	Jang et al. 2022 [44] Torkzaban et al. 2023 [74]	0.94 (CI 0.89–0.98) in total cohort and 0.93 (95% CI: 0.84–0.98). in cohort excluding living donors 0.69 (CI N/A)	132 (53) 34 (11)	NAS ≥ 3 + ≥ 1 point for each category – steatosis, inflammation, ballooning NAS ≥ 3. One point for each category	0.81 se and 0.96 spec in total cohort and 0.85 se and 0.96 spec in cohort excluding living donors N/A
Grey‐scale US index (SWE and HRI) and AST Grey‐scale US index (SWE + HRI) Grey‐scale US index (SWE + HRI) + fibroscan Grey‐scale US index (SWE + HRI) + fibroscan + AST	Kim et al. 2022 [47]	0.81 (CI 0.68–0.90) 0.79 (CI 0.67–0.89) 0.79 (CI 0.66–0.88) 0.80 (CI 0.67–0.89)	60 (25)	NAS ≥ 5	68.0 (46.5–85.1) se and 80.0 (63.1–91.6) 68.0 (46.5–85.1) se and 85.7 (69.7–95.2) spec 72.0 (50.6–87.9) se and 85.7 (69.7–95.2) spec 56.0 (34.9–75.6) se and 97.1 (85.1–99.9) spec
US + scoring system based on AC and SWE	Lee et al. 2021 [50]	0.93 (CI 0.87–0.97)	102 (55)	NAS > 3 + ≥ 1 point for each category – steatosis, inflammation, ballooning	1. 0.93 se and 0.83 spec
Modified Fatty Score (FS) (0–2)– 0 for FS < 7 and the sum of parenchymal echogenicity + gallbladder wall blurring < 3; score 1 for FS ≥ 7 or the latter ≥ 3; score 2 for FS ≥ 7 and the latter ≥ 3 Fatty score (0–8) – 5 US parameters parenchymal echogenicity, far gain attenuation, gallbladder wall blurring, portal veinwall blurring and hepatic vein blurring	Liang et al. 2007 [57]	0.82 (CI N/A) 0.79 (CI N/A)	101 (72)	Brunt criteria with modifications. NASH was defined as the presence of fibrosis (grade 1 or higher) or acinar zone 3 hepatocellular injury with ballooning (grade 2 or higher)	N/A 2 0.72 se and 0.86 spec at cut‐off 3 3 0.81 se and 0.66 spec at cut‐off 7
US‐Fatty liver (mild, moderate, or severe according to the fall in echo amplitude, extent of liver/kidney discrepancy and of echo loss from portal vein)	Petrick et al. 2015 [86]	N/A	513 (164)	Brunt criteria	Mild 0.89 se and 0.45 spec Moderate 0.61 se and 0.70 spec
Total Cholesterol, ALT, Ast/alt ratio, γGT and US showing steatosis (ALT = /> 30)	Pulzi et al. 2011 [65]	0.82 (CI 0.67–0.98)	45 (10)	Brunt criteria. Patients with steatohepatitis or fibrosis	0.70 se and 0.89 spec
Three US parameters; DS, AC and SWS	Sugimoto et al. 2020 [71] Torkzaban et al. 2023 [74]	0.81 (CI 0.71–0.91) 0.87 (CI N/A)	111 (90) 34 (11)	Matteoni classification – type 3 and type 4 were defined as NASH NAS ≥ 3. One point for each category	N/A N/A
Ultrasonographic spleen longitudinal diameter	Tarantino et al. 2009 [72]	0.92 (CI N/A)	83 (40)	Kleiner no further specified NASH definition	0.88 se and 0.95 spec cut‐off 116 mm
AC, LS and DS with BMI DS + AC + BMI	Torkzaban et al. 2023 [74]	1.0.87 (CI N/A) 2.0.72 (CI N/A)	34 (11)	NAS ≥ 3. One point for each category	N/A
Attenuation of the echo amplitude, enlarged splenic diameter and presence of focal fat sparing Attenuation of the echo amplitude and focal fat sparing	Zardi et al. 2011 [79]	N/A	94 (74)	The appearance of steatosis, lobular and hepatocyte ballooning alone or together with Mallory's hyaline bodies or fibrosis	0.74 se and 0.66 spec at cut‐off ≥ 5 0.92 se and 0.75 spec at cut‐off 1
US + SWS, AC, NLV (normalised local variance), age and glucose	Zhao et al. 2023 [80]	0.78 (CI N/A) Validation set	75 (16) total cohort, validation 13	Matteoni classification – type 3 and type 4 were defined as NASH	N/A
US	Saadeh et al. 2002 [70]	None of the radiological features included in the CT, US, or MRI protocols could distinguish patients with the pathologic diagnosis of NASH from steatosis	25 (17)	Type 3 (steatosis, lobular inflammation, hepatocyte ballooning) and type 4 (steatosis, lobular inflammation, hepatocyte ballooning and Mallory's hyaline or fibrosis). Patients with 3 and 4 were categorised as NASH	N/A
*2D‐SW*					
SWE (Liver stiffness)	Jang et al. 2022 [44] Zhou et al. 2022 [81] Imajo et al. 2021 [43]	0.85 (CI 0.78–0.91) 0.88 (CI 0.82–0.95) non‐cirrhosis NASH + differentiating cirrhosis NASH 0.95 (CI 0.89–1.00) AUROC was only given for training and not testing cohort 0.58 (CI 0.45–0.71)	132 (53) 139 total cohort (78 patients had non‐cirrhosis NASH, 17 had cirrhosis NASH) 145 (87)	NAS > 3 + ≥ 1 point for each category – steatosis, inflammation, ballooning Kleiner/Brunt criteria classification to include fibrosis NAS ≥ 4 and at least one in ballooning ≥ 1 and inflammation ≥ 1	0.83 se and 0.075 spec at 6 kPa 0.71 se and 0.99 spec at cut‐off 7.3 kPa Non‐cirrhosis training cohort 0.86 se and 0.98 testing cohort 0.88 se and 0.91 at cut‐off 12.6 kPa in the cirrhosis training cohort and 0.87 se and 0.91 spec in testing cohort N/A
SWS (speed)	Sugimoto et al. 2020 [71] Ozturk et al. 2020 [62] Kim et al. 2022 [49]	0.70 (CI 0.58–0.83) 0.67 (CI 0.55–0.79) 0.78 (CI 0.60–0.97)	111 (90) 116 (24) 25 (13)	Matteoni classification – type 3 and type 4 were defined as NASH. NAS ≥ 5 NAS > 3 the presence of steatosis (> 5% of hepatocytes) and the presence of lobular inflammation and hepatocyte ballooning ≥ 1 point each	N/A Se 0.71 and spec 0.64 cut‐off 8.5 kPA (1.68 m/s) SW speed 0.92 se and 0.58 spec threshold 1.52 m/s
SWD (dispersion slope)	Jang et al. 2022 [44] Lee et al. 2021 [50] Kim et al. 2022 [49] Sugimoto et al. 2020 [71]	0.86 (CI 0.79–0.92) 0.89 (CI 0.82–0.95) 0.83 (CI 0.66–1.00) 0.76 (CI 0.63–0.88)	132 (53) 102 (55) 25 (13) 111 (90)	NAS > 3 + ≥ 1 point for each category – steatosis, inflammation, ballooning NAS > 3 + ≥ 1 point for each category – steatosis, inflammation, ballooning NAS > 3 + the presence of steatosis (> 5%) and the presence of lobular inflammation and hepatocyte ballooning ≥ 1 point each Matteoni classification – type 3 and type 4 were defined as NASH	0.85 se and 0.82 spec at 11.4 (m/s)/KHz 0.86 se and 0.87 spec se 0.69 and spec 0.92 slope 11.05 (m/s)/kHz N/A
*AFRI*					
ARFI	Braticevici et al. 2013 [33] Guzmán‐Aroca et al. 2012 [39]	0.87 (CI 0.78–0.95) 0.90 (CI N/A) Simple steatosis (group A) versus NASH or fibrosis (group B + C)	64 (43) 32 (group B inflammation 18, group C fibrosis 6)	Brunt (Kleiner) Matteoni classification. group 1, steatosis without inflammation or fibrosis; group 2, steatosis with lobular inflammation without fibrosis; group 3, additional presence of ballooning hepatocytes; group 4, presence of Mallory's hyaline and/or fibrosis. The patients were classified into three groups: group A, simple steatosis; group B, inflammation; and group C, fibrosis	ARFI velocity > 1.10 m/s as the cut‐off 0.77 se and 0.72 spec Cut‐off value 1.3 m/s; se 0.85 and spec 0.83

Abbreviations: AC, attenuation coefficient; ALT, alanine aminotransferase; ARFI, acoustic radiation force impulse; AST, aspartate aminotransferase; BMI, body mass index; CI, Confidence Interval; DS, dispersion slope; FLI, fatty liver indicator; HRI, hepatorenal index; kHz, kilohertz; kPa, kilopascals; LS, liver stiffness; m/s, metres per second; NAS, NAFLD activity score; PATT, perihepatic adipose tissue thickness; POCUS, point of care ultrasound; QUS, quantitative ultrasound; Se, sensitivity; Spec, specificity; SW, shear wave; SWE, shear wave elastography; US, ultrasound.

Three studies used a model called the ultrasound fatty liver indicator (US‐FLI) developed by Ballestri et al., based on five ultrasound parameters – liver/kidney contrast, posterior attenuation of ultrasound beam, vessel blurring, gallbladder wall blurring, diaphragm blurring and areas of focal sparing – to give a score of 2–8 [25, 69, 84]. Nelson et al. reported an AUROC of 0.93 (CI 0.83–0.97) when the US‐FLI ≥ 5 to differentiate normal versus NASH [84]. Ballestri et al. demonstrated an AUROC of 0.80 (CI 0.676–0.916) in their study on patients with a NAS ≥ 5. It scored high risk for patient selection as it excluded insulin‐treated type 2 diabetes patients [25]. A further study using point of care ultrasound (POCUS) also used US‐FLI; they demonstrated an AUROC of 0.78 [69].

Seven studies in total evaluated SW, reporting low to high AUROCs: 0.58 to 0.95 [43, 44, 49, 50, 62, 71, 81]. The highest AUROC was reported in a study by Zhou et al., who used the Brunt classification of NASH and therefore included fibrosis in this, and the AUROC of 0.95 was seen in a subset of the population with cirrhosis. It suffered from bias due to including biopsied controls in its cohort; however, it did use 5‐fold cross‐validation for its results [81].

We found two studies reporting on ARFI alone; these report good AUROC of 0.87 and 0.90 and use similar cut‐offs (ARFI velocity > 1.10 m/s and 1.3 m/s). They both report on high sensitivities and specialities [33, 39].

##### 3.7.2.2 Vibration‐Controlled Transient Elastography (VCTE), CAP and MASH

Within the 11 studies that evaluated TE, AUROC ranged from 0.35 to 0.82 (Table 4). The lowest scoring was for Park et al. [63]. It included patients with borderline NASH or definite NASH as per the NASH CRN within its pathological definition. The highest AUROC was shared by two studies: firstly by Salvati et al. for a model combining TE and BMI score [66], and secondly by Eddowes et al., which categorised NASH based on the presence of lobular inflammation and hepatocyte ballooning. Within the same study, when they also report on NAS ≥ 5, the AUROC was 0.74 (CI 0.59–0.89) [31]. There was a wide range of thresholds used for TE from > 5.3 to > 9.9 kpa used, and five studies did not report on the threshold used.

**TABLE 4:** Diagnostic accuracy of VCTE and CAP in MASH diagnosis.

Method/model used	Authors and year	AUROC (95% CL)	Population number (MASH)	Path grading	Sensitivity and specificity
VCTE	Alsaqal et al. 2021 [23] Eddowes et al. 2018 [31] Kim et al. 2022 [47] Imajo et al. 2021 [43] Lee et al. 2022 [52] Lee et al. 2020 [54] Troelstra et al. 2021 [76] Park et al. 2017 [63] Lee et al. 2016 [51] Salvati et al. 2021 [66] Eilenberg et al. 2021 [32]	0.77 (CI N/A) 0.82 (CI 0.69–0.94) NASH versus simple steatosis + 0.74 (0.59–0.89) NAS < 5 versus NAS ≥ 5 0.69 (CI 0.55–0.80) 0.56 (CI 0.45–0.66) 0.72 (C: 0.65–0.78) 0.70 (CI 0.61–0.78) 0.73 (CI N/A) 0.35 (CI 0.22–0.49) 0.75 (CI 0.68–0.82) 0.75 (CI N/A) 0.64 (PP group) and 0.63 (ITD group)^a^ (CI N/A)	68 (53) 50 (38) 60 (25) 145 (87) 251 (117) 130 (70) 37 (22) 104 (72) with biopsy but only 94 with valid TE 183 (94) 98 (77) 170 ITD^a^ and 154 PP (103)	NAS > 3 grade 1 for each of the three features Presence of lobular inflammation and hepatocyte ballooning + NAS for grading NAS ≥ 5 NAS ≥ 4 and at least one in ballooning ≥ 1 and inflammation ≥ 1 NAS ≥ 5 NAS ≥ 3; > 5% of hepatic steatosis and inflammation were seen with hepatocyte ballooning SAF NAS ≥ 3 Presence of steatosis and inflammation with ballooning Hepatic steatosis and inflammation with hepatocyte injury SAF	0.82 se and 0.73 spec cut‐off > 5.3 kPa N/A 0.48 (0.28–0. 69) se and 0.89 (0.73–0.97) spec 0.44 (0.24–0.65) se and 0.97 (0.85–1.0) spec Se 0.78 and spec 0.63 cut‐off 7.7 kPa N/A 0.64 se and 0.87 spec at 9.9kpa cut‐off 0.61 se and 0.59 spec cut‐off of 5.6 kPa Se 0.86 and spec 0.58 cut‐off > 7 kPa Se 0.73 and 0.75 spec cut‐off 5.75 kPa 0.54 se and 0.46 spec cut‐off 6 kPa (PP^a^) and 0.54 se and 0.46 spec cut‐off of 6 kPa (ITD group)^a^
Fibroscan + AST index	Kim et al. 2022 [47]	0.74 (0.61–0.85)	60 (25)	NAS ≥ 5	0.44 (0.24–0.65) se and 0.97 (0.85–1.0) spec
VCTE‐LS (fibroscan) and CAP	Imajo et al. 2021 [43]	0.71 (CI 0.66–0.8)	145 (87)	NASH was defined as NAS ≥ 4 and at least one in ballooning ≥ 1 and inflammation ≥ 1	N/A No cut‐off given
CLA model – CAP values greater than 250 dB/m, LS > 7 kPa, ALT level > 60 IU/L, hypertension, current smoker and presence of metabolic syndrome	Lee et al. 2016 [51]	0.81 (CI 0.72–0.88) The bootstrap method, average value of 0.83 (CI 0.74–0.89)	183 (94)	NASH was defined as the presence of steatosis and inflammation with ballooning regardless of fibrosis	Model N/A
FAST score	Lee et al. 2022 [52] Eilenberg et al. 2021 [32] per protocol (PP) analysis, excluding unreliable results as well as an intent‐to‐diagnose (ITD) analysis including failed measurements	0.75 (CI 0.69–0.81) 0.78 (CI N/A)	251 (117) 170 ITP and 154 PP (103)	NAS ≥ 5 SAF	Se 0.80 and spec 0.58 Fast cut‐off 0.48 0.59 se and 0.90 spec with a cut‐off 366 (PP group) and se 0.62 and 0.87 spec (ITD group)
TE and BMI score	Salvatia et al. 2021 [66]	0.82 (CI N/A)	98 (77)	NASH was defined as hepatic steatosis and inflammation with hepatocyte injury (e.g., ballooning)	0.75 se and 0.80 spec cut‐off of 154.56 kPa•kg/m^2^ 0.83 se and 0.83 spec with the cut‐off of 98.11 kPa•kg/m^2^
Bayesian approach – CK‐18 levels, hydrogen breath test and TE	Yilmaz et al. 2014 [78]	N/A	235 (135)	NAS ≥ 5	N/A
*CAP*					
CAP	Garteiser et al. 2021 [38] Imajo et al. 2021 [43] Trowell et al. 2021 [75] Troelstra et al. 2021 [76] Lee et al. 2016 [51] Eilenberg et al. 2021 [32]	0.69 (CI 0.59–0.79) 0.71 (CI 0.62–0.80) 0.80 (CI N/A) Validation 0.65 (CI N/A) 0.74 (CI 0.67–0.82) 0.76 (PP group)^a^ and 0.76 (ITD group)^a^ (CI N/A)	152 (30) 145 (87) 217 (68) total cohort (Training 151 and validation 66) 37 (22) 183 (94) 170 ITP and 154 PP (103)	FLIP NAS ≥ 4 and ballooning ≥ 1 and inflammation ≥ 1 NAS ≥ 3 Presence of steatosis where hepatocellular ballooning grade was ≥ 1 and where lobular inflammation grade ≥ 1 was present Presence of steatosis and inflammation with ballooning regardless of fibrosis SAF	Se of 0.86 (0.71–0.95) and Sp of 0.47 (0.35–0.59), at a cut‐off of 318 dB/m N/A Validation 0.82 se and 0.80 spec cut‐off 301 dB/m 0.77 se and 0.53 spec cut‐off 336 dB/m CAP > 250 dB/m se 0.96 and spec 0.49 0.80 se and 0.62 spec at cut‐off of 323.5 dB/m + 0.81 se and 0.60 spec at cut‐off of 323.5 dB/m
CAST – CAP + AST	Imajo et al. 2023 [42]	0.77 (CI 0.67–0.85) Validation cohort 0.69 (CI 0.62–0.76) In the US cohort	169 (98) validation cohort 234 (117) USCD cohort	NAS ≥ 4	N/A
CIR score – CAP, HOMA and insulin resistance	Macias et al. 2022 [59]	0.93 (CI 0.87–0.99) Testing cohort	155 (28)	SAF	N/A

Abbreviations: ALT, alanine aminotransferase; AST, aspartate aminotransferase; BMI, body mass index; CAP, controlled attenuated parameter; CI, confidence interval; CK‐18, cytokeratin 18; dB/m, decibels per meter; FAST, Fibroscan and AST; HOMA, homeostatic model for assessment of insulin resistance; ITD, intent‐to‐diagnose; kg/m^2^, kilograms per metre squared; kPa, kilopascals; kPa, kilopascals; LS, liver stiffness; NAS, NAFLD activity score; PP, per protocol; SAF, steatosis activity and fibrosis score; Se, sensitivity; Spec, specificity; TE, transient elastography.

^a^ Per protocol (PP) analysis excluding unreliable results and intent‐to‐diagnose (ITD) analysis based includes failed measurements.

One study by Imajo et al. compared SW on 2D ultrasound and Fibroscan. Fibroscan was reported to have a 0.56 AUROC for NASH, and 2DSW was reported to have an AUROC of 0.58 (CI 0.45–0.71). In the same study, the authors also combined Fibroscan with CAP for NASH diagnosis, which improved the AUROC to 0.71 (CI: 0.66–0.8) [43]. There was no other paper using this combination. Kim et al. also combined TE with US features of 2D‐SW and HRI for diagnosis of NAS ≥ 5. This gave an AUROC of 0.79 (CI 0.66–0.88); AST was added to this model, which relived a similar AUROC of 0.80 (CI 0.67–0.89) [47].

Eight studies looked at CAP and MASH diagnosis with a range of AUROC from 0.65 to 0.93 (Table 4). The best was for the CIR score, which combined CAP score, HOMA and insulin resistance by Macias et al. in their testing cohort but does not give AUROC for their validation cohort [59]. The lowest AUROC (0.65) was by Troelstra et al. for differentiating steatosis versus NASH using CAP alone. They used a higher cut‐off of > 336 m/s than used in other studies [76]. A cut‐off > 301 m/s by Trowell et al. give the best AUROC of 0.80 for CAP alone [75].

#### 3.7.3 Other Imaging and MASH

There were five CT studies; these showed a range of AUROC from < 0.60 to 0.94 (Table 5). In the 0.94 study, this included patients with borderline NASH, and this was in patients without suspicion of fibrosis determined by low hyaluronic acid levels. When looking at patients with suspicion of fibrosis, the AUROC was 0.60 in the same study [61]. The second highest AUROC was 0.90 when looking at the mean value of positive pixels (MPP) below 54.0 in a study by Dichtel et al. [29] This also reported good sensitivity (100%) and specificity (100%).

**TABLE 5:** Diagnostic accuracy of other imaging methods in MASH diagnosis.

Method/model used	Authors and year	AUROC (95% CL)	Population number (MASH)	Path grading	Sensitivity and specificity
CT					
Entropy MPP	Dichtel et al. 2023 [29]	0.88 (CI 0.62–0.76) 0.90 (CI 0.62–0.76)	16 (10)	NAS ≥ 1 in each category	0.8 se (CI 0.50–1.00) and 1.0 spec 95% CI: 67%–100%) entropy values below 4.73 1.0 (0.50–1.00) se and 1.0 (67%–100%) spec MPP values below 54.0
Liver‐spleen HU Liver HU	Kim et al. 2023 [46]	0.82 L‐S HU distinguishing NAS > 4 versus < 4 (CI N/A) + 0.75 for NAS < 3 versus NAS ≥ 3 0.86 (CI N/A) Liver HU NAS > 4 versus < 4 + 0.70 (CI N/A) for NAS < 3 versus NAS ≥ 3 In patient group with low grade fatty liver disease	142 (6)	NAS > 4 G1 = Group NAS < 3; G2 = Group NAS 3–4 G3 = Group NAS ≥ 4	0.71 se and 0.71 spec cut‐off 3 for NAS ≥ 3 and L–S HU value, with a cut‐off value of −1, NAS ≥ 5 with 1.0 se and 0.71 spec 2 N/A
CT parameters	Lubner et al. 2023 [58]	< 0.60 for all individual CT features and subjective assessments	186 (87)	Patients were considered to have NASH if hepatic steatosis and any lobular inflammation or hepatic ballooning was identified	N/A
Non‐contrast enhanced CT 1 NASH prediction model for patients without suspicion of fibrosis – 12 parameters 2 NASH prediction model for patients with a suspicion of fibrosis using 2 parameters 2 Fibrosis was prediction by elevated levels of serum hyaluronic acid	Naganawa et al. 2018 [61]	0.94 (CI N/A) In patients without suspected fibrosis (texture parameter mean without filter and skewness with a 2‐mm filter) 0.60 (CI N/A) in patients with suspicion of fibrosis (the mean without filtration and kurtosis with a 4‐mm filter) Validation cohort	35 (NASH number N/A) validation	NAS was ≥ 3	1.0 se and 0.92 spec 1.0 se and 0.31 spec
CT parameters	Saadeh et al. 2002 [70]	N/A. None of the radiological features could distinguish patients with the pathologic diagnosis of NASH from steatosis	25 (17)	Type 3 (steatosis, lobular inflammation, hepatocyte ballooning) and type 4 (steatosis, lobular inflammation, hepatocyte ballooning and Mallory's hyaline or fibrosis). Patients with types 3 and 4 were categorised as NASH	N/A
*Scintigraphy*					
Scintigraphy 9mTc‐phytate scintigraphy	D'Avila da Silva et al. 2021 [67] Kikuchi et al. 2009 [45]	N/A; the results did not reach the desired levels 0.82 (CI N/A)	1.61 (49) 37 (29)	Presence of steatosis associated with ballooning and inflammatory infiltrate NAS ≥ 5	N/A Se of 1.0 and 0.75 spec with a cut‐off point of 2.93, the liver/spleen uptake ratio

Abbreviations: CI, Confidence Interval; L‐S HU, liver spleen Hounsfield unit; MPP, mean value of positive pixels; NAS, NAFLD activity score; Se, sensitivity; Spec, specificity.

There were scintigraphy studies [45, 67]. In the study by Kikuchi et al., scintigraphy images were obtained after the infusion of radiopharmaceutical ^99m^Tc‐phytate in liver/spleen, spleen/heart, liver/heart ratios and ^99m^Tc‐isonitrile liver/heart ratio. The authors saw a reduction in spleen/uptake ratios with NASH versus steatosis [45]. This was not replicated in a later study by D'Avila da Silva et al., which did not show any difference in liver/spleen uptake between groups [67].

## 4 Clinical Trials

We identified eight clinical trials in our search (Supporting Information) [82, 91, 92, 93, 94, 95, 96, 97]. One is already finished and included in our paper [82]. They evaluate 2D SW, MRI liver multiscan, PET, ultrasound and spin‐lock MRI. Four trials have finished recruiting and two have yet to start recruiting. One large trial, with a target recruitment of 450 patients, called the LITMUS imaging study is evaluating Liver*MultiScan*, MRE, MRI, MRI PDFF, 2D SWE, pSWE and TE. Its primary outcome is the diagnosis of fibrosis, but has a MASH diagnosis in its secondary outcome [96].

## 5 Discussion

This study critically reviewed 69 papers reporting an imaging test or an imaging‐based score for the diagnosis of MASH. We searched a range of databases to include grey literature and trial registers. We also report on the use of pathology definitions to make it transparent what was being tested and included all imaging tools found rather than focusing on one method to give a more accurate perspective of available tests. We found that ultrasound and MRI are the most investigated imaging modalities and showed the most promise in our results for the diagnosis of MASH. Other modalities, including CT, TE, CAP and scintigraphy, had more variable results with limited studies and, in the case of TE, yielded better results when combined with other imaging modality. Ultrasound and MRI carry no radiation, unlike CT. Compared to MRI, ultrasound has a lower cost and can be used in patients with claustrophobia and metal implants/pacemakers that are not MRI‐safe. However, adequate ultrasound imaging is restricted in obese patients.

Within the MRI studies, MRE studies especially showed good AUROCs for distinguishing between NASH and simple steatosis. However, Imajo et al. reported an AUROC of only 0.57 (CI: 0.47–0.67) in their study [43]. The highest AUROC of 0.93 was by Chen et al. Although the study did show that there was higher liver stiffness in patients with NASH without fibrosis over patients with simple steatosis, there was the most liver stiffness in those with fibrosis. The study used the Brunt criteria, which include fibrosis within its pathological definition, which may account for the higher AUROC [23]. This would suggest that, like any elastography method due to liver stiffness, MRE may yield better results in patients with MASH and fibrosis than in patients with MASH without fibrosis. However, other studies assessing MRE for identifying cases with MASH and fibrosis ≥ 2 suggest lower results, with an AUROC of only 0.66 in the study by Imajo et al. [43], and Costa‐silva et al. report an AUROC of 0.79 for MRE using NAS ≥ 5, a NASH definition that does not include fibrosis [27]. SPIO has good results with AUROCs of 0.79 and 0.87, but there are safety concerns regarding this that limit its use [68, 73]. Two studies reported on the use of MRS, but the ability of MRS, high cost and long length of scans limit its use in clinical practice [20, 48]. Kim et al. found NASH patients had higher levels of Alanine (Ala) (*p* < 0.001) and Lactate (Lac) + Triglyceride (TG) (*p* < 0.001) than simple steatosis or controls. They found that Ala concentration had the best sensitivity of 100% and a specificity of 100% with a cut‐off of > 16.04% (AUROC 1.0) [48]. The second larger study on MRS showed a lower 0.71 AUROC [20]. These studies did have different study designs. Abrigo et al. looked at biochemical changes with 31‐Phopshate‐MRS (^31^P‐MRS) and found that NASH patients had a decreased alpha nucleotide triphosphate (α‐NTP)/total phosphate (TP) (*p* < 0.001) ratio. An α‐NTP/TP cut‐off of 16.36% gave 91% sensitivity, and a cut‐off of 10.57% gave 91% specificity for NASH [20]. The Abrigo et al. study contained 151 patients (95 NASH) compared to 32 patients (11 NASH) in the Kim et al. study. The studies also used different pathology definitions [20, 48]. When evaluating MRI alone, Leporq et al. reported on a decrease in magnetic susceptibility in NASH leading to an AUROC of 0.91 [55] but we found no other studies on this technique to check repeatability. Additionally, Kim et al. report on a multiparametric MRI index obtaining an AUROC of 0.88, but again, [82] we found no other study on this model.

AUROCs > 0.90 were seen in a total of 7/27 ultrasound‐based studies, [35, 39, 44, 50, 72, 81, 84] although one was for SW in a group of cirrhotic NASH patients [81]. This contrasts with MRI; despite more studies within this review, only two had an AUROC > 0.90 from our papers [28, 55]. Four of these studies contained SW in its model [35, 44, 50, 81]. SW itself had variable results ranging from 0.58 to 0.95, but the latter study was in cirrhotic NASH. This study achieved an AUROC of 0.88 for non‐cirrhotic NASH in 2D‐SWE and 0.95 in cirrhotic NASH, demonstrating elastography achieving better outcomes with more advanced fibrosis [81]. US based combination scores yielded the best results, such as the NASH pentagon by Funada et al. combining ultrasound parameters. The NASH pentagon achieved an AUROC of 0.94; the study did include fibrosis in their pathological diagnosis but did not report on any sensitivity and specificity levels [35]. However, Jang et al., with their study, also achieved an AUROC of 0.94 for a US risk score. They used the FLIP criteria. They also report good sensitivity (0.81) and specificity (0.96) with this study [44]. Three studies reported on US‐FLI; this utilises five ultrasound parameters to give a diagnosis of NASH [25, 69, 84]. Nelson et al. achieved a high AUROC for differentiating NASH versus no NASH, but when comparing steatosis versus NASH, the AUROC was 0.65 (95% CI, 0 0.56–0.72) [84]. Ballestri et al. demonstrated a high AUROC of 0.80 when NAS ≥ 5 and a US‐FLI score of 4 or lower suggested the absence of NASH (negative predictive value, 88%; sensitivity, 91% but specificity was 46%) [25]. The third study using POCUS and US‐FLI found an AUROC of 0.78 and when US‐FLI ≤ 3 and a sensitivity of 1.0 for ruling out NASH. At a cut‐off ≥ 6, US‐FLI had 63% specificity in the study. They used the NAS score but did not comment on the actual number used [69]. Overall, the studies suggest the score is better for ruling out NASH rather than differentiating it from steatosis. One of the disadvantages with this score is that one of the parameters is gallbladder blurring; therefore, the patient must have a gallbladder to evaluate. We found only two studies on ARFI that showed good AUROC, sensitivities and specificities [33, 39]. More studies on this technique are needed.

The studies unfortunately contained major sources of heterogenicity, which precluded a meta‐analysis. Firstly, like other similar systematic reviews on diagnosis of MASH, we found our studies used heterogenous definitions of MASH, which makes comparisons between tests difficult. The definition used may cause better outcomes. For example, if fibrosis was included in the definition as with Brunt criteria, which can diagnose MASH based on the fibrosis stage alone, then elastography methods may have an advantage here. As our results illustrate, the best AUROC in MRE and 2D‐SWE are in studies using the Brunt criteria [28, 81]. The high scores may reflect the diagnosis of fibrosis rather than the inflammatory component. These would likely create different outcomes if different pathological definitions were used. Secondly, imaging techniques were often different even within the same modularity, especially ultrasound, which utilised several different combinations of scores. Thirdly, different thresholds were used. Studies often used the Youden index to obtain the optional threshold for their study, with varying results between papers. For the 11 studies that looked at transient elastography, 7 documented the thresholds used within the text, and each of these studies used a different threshold [23, 32, 51, 52, 63, 66, 76]. Within the 11 papers that reported on 2D‐MRE, 6 commented on the threshold used and only two studies used the same threshold [23, 28]. Additionally, 5/11 MRE studies did not report sensitivity or specificity data [22, 43, 54, 56, 87], and there was only a maximum of two studies using the same defined pathology score [27, 63, 87, 88]. Thus, these variations precluded a meta‐analysis.

Our study found that the most promising imaging tools are MRI techniques or ultrasound‐based scores and confirmed there is potential for MASH diagnosis. Within the US studies, there are specific US parameters that have been found to be significant when diagnosing MASH, such as hepatic vessel blurring, attenuation coefficient and hepato‐renal index. Further studies could focus on these identified parameters. Additionally, for future studies, there needs to be a standard pathological definition of MASH used to compare studies or the availability of digital histology images to allow re‐scoring of biopsies. Furthermore, more validation studies are needed, which will help minimise the bias found in studies and prove the repeatability of the tests/scores. Many of these papers have developed a model that has not been tested elsewhere. Validating in larger studies can be difficult to undertake due to costs, time and availability of participants or data. Work is required in this area to make it easier for researchers to validate their studies. A new system, perhaps a large, easily accessible data pool, may help this. Without larger validation studies, there is no clear imaging tool or score currently available to diagnose MASH reliably.

### 5.1 Study Limitations

Firstly, many of the studies suffered from bias. Introduction of a control group in the results, as seen in three of the papers, [20, 41, 44] may affect the accuracy of tests and does not reflect real‐world testing of at‐risk groups. Only 14 studies had pre‐set cut‐offs or offered a validation for their index test; thus, the reminders were deemed at high risk of bias for the index test section within the tool [34, 36, 37, 42, 53, 59, 60, 61, 69, 70, 75, 81, 86, 88]. This is understandable when assessing a new diagnostic test but will cause bias, particularly when cut‐offs are adjusted to obtain higher sensitivity and specificity levels. For this systematic review, a comprehensive search was conducted, but studies without a clear report of liver biopsy or MASH diagnosis could have been omitted. Secondly, we did not analyse our planned secondary outcome of reviewing papers in relation to the components of the NAS score. This was only available in a limited selection of papers, and therefore felt not as valuable to the overall focus of the paper. However, this could be considered for future work.

## 6 Conclusion

With advances in the treatment of MASH and studies showing MASH resolution resolves fibrosis and improves survival, then a focus on diagnosis of MASH rather than fibrosis is important. If we focus on fibrosis, then we would be missing an important group of patients with MASH and little/no fibrosis that could go on to develop fibrosis in the future and are currently subject to increased risk of cancer and lower survival than the general population. Imaging tools have been studied in the diagnosis of MASH, and there has been some progress made, especially ultrasound and MRI techniques. However, larger validation studies and an easier pathway for validation are needed.

## Author Contributions

J.C. prepared the study protocol, which was reviewed and edited by R.B., J.S.B., R.L., M.A. and J.F.D. J.C. and R.B. performed the searches and screening of abstracts and titles, as well as the bias review of papers. J.C. drafted the manuscript, which was reviewed and edited by R.B., J.S.B., R.L., M.A. and J.F.D.

## Conflicts of Interest

J.S.B. reports honoraria and research funding by Intelligent Ultrasound (IU) Company. J.C. reports funding of her Medicine Doctorate by Intelligent Ultrasound Company. J.F.D. reports receiving consulting fees from IU and honoraria from GE ultrasound. IU and GE had no involvement in study design; in the collection, analysis and interpretation of data; in the writing of the report; and in the decision to submit the article for publication. R.B., R.L. and A.M. declare no conflicts of interest.

## Supporting information


Data S1.

## Data Availability

Data for this systematic review are provided in Supporting Information or otherwise available at reasonable request.
