# Supplementary material for: Accuracy of Non‐Invasive Imaging Techniques for the Diagnosis of MASH in Patients With MASLD: A Systematic Review
Source: Liver Int. 2024 Oct 14;45(4):e16127. doi: 10.1111/liv.16127 (PMC11891385; doi:10.1111/liv.16127)
Supplement: Supplementary file 1 — Data S1. [file LIV-45-0-s001.docx]

**Accuracy of non-invasive imaging techniques for the diagnosis of metabolic dysfunction associated steatohepatitis (MASH); A systematic review**

**Jennifer Cathcart,^1,2^ Rachael Barrett,^1^ James S Bowness James,^3,4^ Ashis Mukhopadhya,^2^ Ruairi Lynch,^1^ and John F Dillon^1^**

**1 University of Dundee**

**2 Gastroenterology department, Aberdeen Royal infirmary**

**3 University College London Hospitals NHS Foundation Trust**

**4 University College London**

Contents

1. Search terms
2. Prisma flow chart
3. QUADRAS bias tool
4. GRADE certainty of evidence
5. Demographics of papers
6. Imaging studies and high risk MASH (NAS ≥ 4 and F2)
7. Clinical Trials

Search terms

Pubmed

("Non alcoholic steatohepatitis" OR "nonalcoholic steatohepatitis" OR "NASH" OR "Metabolic dysfunction associated steatohepatitis" OR "MASH") AND (imaging[Title/Abstract] OR "computerised tomography"[Title/Abstract]  OR CT[Title/Abstract]  OR "magnetic resonan*"[Title/Abstract]  OR MRI[Title/Abstract]  OR elastrography[Title/Abstract]  OR fibroscan[Title/Abstract]  OR ultraso*[Title/Abstract]  OR ultrasonography[MeSH Terms] OR "Diagnostic Imaging"[MeSH Terms] OR "nuclear medicine"[Title/Abstract]  OR "acoustic radiation force impulse"[Title/Abstract]  OR "liver scintigraphy"[Title/Abstract]  OR “shear wave”[Title/Abstract] ) AND (sensitiv* OR specific* OR sensitivity and specificity[MeSH Terms] OR "ROC" OR "AUROC" OR "area under the receiver operating" OR accura* OR "likelihood" OR "predict*" OR "ratio") AND (biops* OR histolog* OR pathology OR histopathology OR "NASH-CRN")

Embase

1 ("Non alcoholic steatohepatitis" or "nonalcoholic steatohepatitis" or "NASH" or "Metabolic dysfunction associated steatohepatitis" or "MASH").af. 53443

2 (imaging or "computerised tomography" or CT or "magnetic resonan*" or MRI or elastrography or fibroscan or ultraso* or ultrasonography or "nuclear medicine" or "acoustic radiation force impulse" or "liver scintigraphy" or "shear wave").ab,ti. 2788402

3 exp *diagnostic imaging/ 59802

4 (sensitiv* or specific* or "ROC" or "AUROC" or "area under the receiver operating" or accura* or "likelihood" or "predict*" or "ratio").af. 11112564

5 exp *"sensitivity and specificity"/ 2976

6 (biops* or histolog* or pathology or histopathology or "NASH-CRN").af. 4690347

7 2 or 3 2805310

8 4 or 5 11112564

9 1 and 6 and 7 and 8 2194

Cochrane

#1 ("Non alcoholic steatohepatitis" or "nonalcoholic steatohepatitis" or "NASH" or "Metabolic dysfunction associated steatohepatitis" or "MASH") 3196

#2 ((imaging or "computerised tomography" or CT or (magnetic NEXT resonan*) or MRI or elastrography or fibroscan or ultraso* or "nuclear medicine" or "acoustic radiation force impulse" or "liver scintigraphy" or "shear wave")):ti,ab,kw 212214

#3 MeSH descriptor: [Ultrasonography] explode all trees 17668

#4 MeSH descriptor: [Diagnostic Imaging] explode all trees 63209

#5 (sensitiv* or specific* or "ROC" or "AUROC" or "area under the receiver operating" or accura* or "likelihood" or predict* or "ratio") 459997

#6 MeSH descriptor: [Sensitivity and Specificity] explode all trees 19955

#7 (biops* or histolog* or pathology or histopathology or "NASH-CRN") 130727

#8 (#1) AND (#2 OR #3 OR #4) and (#5 OR #6) and (#7) 202

 CINAHL

TX ( ("Non alcoholic steatohepatitis" OR "nonalcoholic steatohepatitis" OR NASH OR "Metabolic dysfunction associated steatohepatitis" OR MASH) ) AND TI ( (imaging OR "computerised tomography" OR CT (OR magnetic resonan*) OR MRI OR elastrography OR fibroscan OR ultraso* OR "nuclear medicine" OR "acoustic radiation force impulse" OR "liver scintigraphy" OR "shear wave" OR (MH Ultrasonography) OR (MH diagnostic imaging) ) AND AB ( (imaging OR "computerised tomography" OR CT (OR magnetic resonan*) OR MRI OR elastrography OR fibroscan OR ultraso* OR "nuclear medicine" OR "acoustic radiation force impulse" OR "liver scintigraphy" OR "shear wave" OR (MH Ultrasonography) OR (MH diagnostic imaging) ) ( (sensitiv* OR specific* OR ROC OR AUROC OR "area under the receiver operating" OR accura* OR likelihood OR predict* OR ratio (MH sensitivity and specificity) ) AND TX ( (biops* OR histolog* OR pathology OR histopathology OR "NASH-CRN") )

Clinical trials. Gov

NASH and Diagnostic Imaging

WHO

Non-alcoholic steatohepatitis and Imaging

Open grey

Non-alcoholic steatohepatitis and Imaging

ACM digital

*[[Full Text: "non alcoholic steatohepatitis"] OR [Full Text: "nonalcoholic steatohepatitis"] OR [Full Text: "metabolic dysfunction associated steatohepatitis"]] AND [[Abstract: imaging] OR [Abstract: "computerised tomography"] OR [Abstract: ct] OR [Abstract: "magnetic resonan*"] OR [Abstract: mri] OR [Abstract: elastrography] OR [Abstract: fibroscan] OR [Abstract: ultraso*] OR [Abstract: ultrasonography] OR [Abstract: "diagnostic imaging"] OR [Abstract: "nuclear medicine"] OR [Abstract: "acoustic radiation force impulse"] OR [Abstract: "liver scintigraphy"] OR [Abstract: "shear wave"]] AND [[Full Text: sensitiv*] OR [Full Text: specific*] OR [Full Text: "roc"] OR [Full Text: "auroc"] OR [Full Text: "area under the receiver operating"] OR [Full Text: accura*] OR [Full Text: "likelihood"] OR [Full Text: "predict*"] OR [Full Text: "ratio"]] AND [[Full Text: biops*] OR [Full Text: histolog*] OR [Full Text: pathology] OR [Full Text: histopathology] OR [Full Text: "nash-crn"]]*

**Prisma flow chart**

**
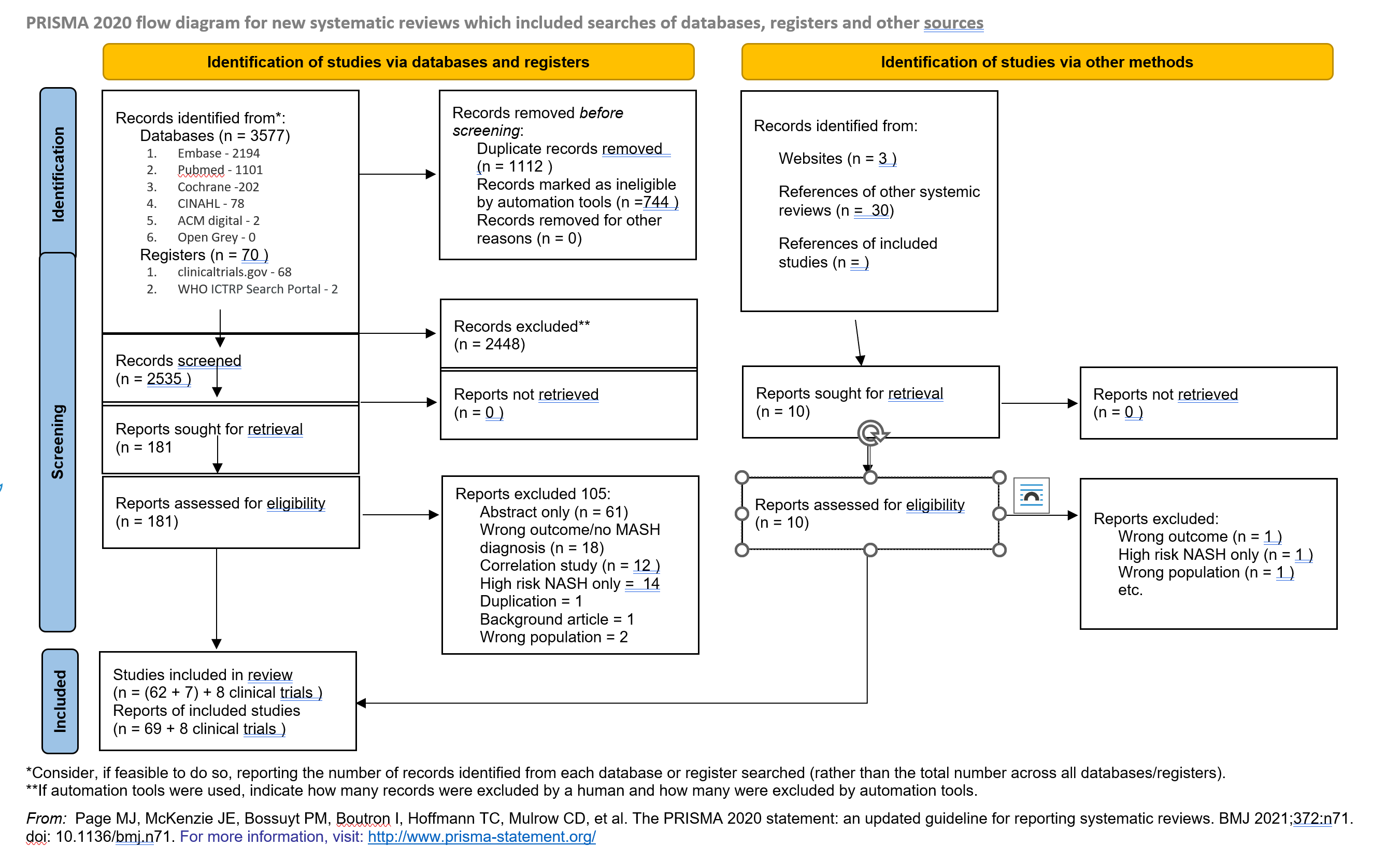
**

**QUADRAS Bias tool**


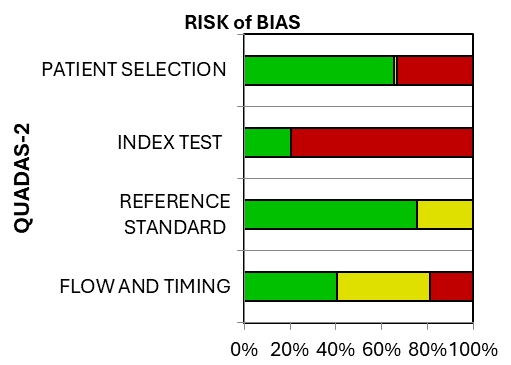

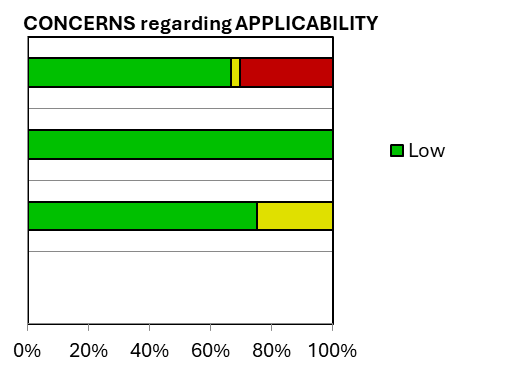

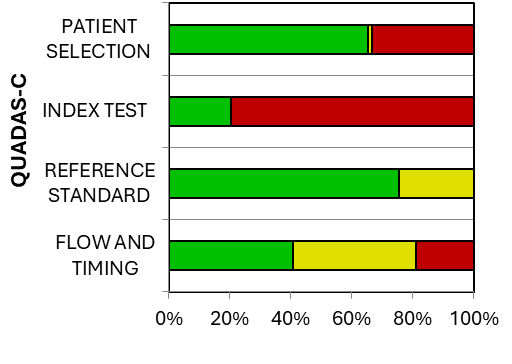


**GRADE certainty of evidence**

| Imaging test | GRADE |
| --- | --- |
| MRE (11 studies) | moderate |
| MRI PDFF (7 studies) | very low |
| Ultrasound (20 studies excluding ARFI, SWE, TE and CAP) | low |
| ARFI (2 studies) | Very low |
| SWE studies (6 studies) | moderate |
| TE (11 studies) | low |
| CAP (6 studies) | moderate |
| CT (5 studies) | moderate |
| Scintigraphy (2 studies) | very low |

**Demographics Table**

| **Year** | **Authors** | **Country** | **Recruiting centres** | **Age (mean)** | **Gender** | **Race** | **BMI (mean kg/m2)** |
| --- | --- | --- | --- | --- | --- | --- | --- |
| 2014 | Abrigo et al (20) | **Hong Kong** | single centre | 47 in control cohort  50 non-NASH NAFLD cohort  51 NASH chort | 11 males (57.0%) in control cohort  18 males (10%) non-NASH cohort  51 males (11%) NASH cohort | n/a | 22.2 controls 26 non-NASH 28.6 NASH |
| 2021 | Manasra et al (21) | **Jordan** | single centre | 34 | females (36/45) 73.5% | n/a | 43 |
| 2020 | Allen et al (22) | **USA** | single centre | 45 | 141 (81%) females | n/a | 46 NASH and 45 non-NASH |
| 2021 | Alsaqal et al (23) | **Sweden** | single centre | 54.5 in total cohort and NASH 53.5 | 40/68 men (58.8%) in total cohort and NASH 32/53 (60.4%) men | 62 (91.2%) Caucasian in total cohort and 47 (88.7%) NASH Caucasian | 30.8 total cohort and NASH 31.2 |
| 2020 | Amorim et al (24) | **Brazil** | single centre | 57.5 in total cohort and 57 NASH cohort | 41/56 (73.2%) female in total cohort and NASH 29/38 (76%) female | n/a | 32.5 total cohort |
| 2012 | Ballestri et al (25) | **Italy** | single centre | 45.1 total cohort and NASH 45.7 | total 37 (69.8%) males and NASH 24 (68.6%) males | n/a | 29.2 NAFLD and 30 NASH |
| 2014 | Bastati et al (26) | **Germany** | single centre | Total cohort 55 mean females age and 56 mean male age | 36 (44%) females' total cohort | n/a | 28.5 |
| 2011 | Chen et al et al (28) | **USA** | single centre | 51.5 | 83% (48 of 58) female | n/a | 38.3 |
| 2018 | Costa-silva at al (27) | **Brazil** | single centre | total cohort 53.8 | 42/49 (85.7%) female | n/a | 32.2 |
| 2023 | Dichtel L.E et al (29) | **USA** | single centre | 57 | 6/16 (38%) male | n/a | 37.5 |
| 2021 | Dzyubak B et al (30) | **USA** | single centre | 47 | 83% female | n/a | 47 |
| 2018 | Eddowes et al (31) | **UK** | 2 centres | 54 NAFLD | 28/50 (56%) male | 43/50 (86%) Caucasian | 33.6 |
| 2021 | Eilenberg et al (32) | **Austria** | single centre | ITP group 42.0 and NASH group 45.0 | ITP 170 (64.7%) female and NASH 29/103 (28%) female | n/a | 44.4 total ITP cohort and NASH cohort 45.2 |
| 2013 | Braticevici et al (33) | **Romania** | single centre | NASH 51 | 23/43 (53.5%) NASH Female | n/a | NASH male 29.55 + NASH female 28.81 |
| 2012 | Francque et al (34) | **Belgium** | single centre | 43.5 in total cohort and  42.9 in validation cohort | 66.3% female in the total cohort and 66.4% female in validation cohort | n/a | 38.9 total cohort  39.7 in validation cohort |
| 2023 | Funada et al (35) | **Japan** | single centre | 55.1 | 46/107 (42.9%) female | n/a | 26.8 |
| 2016 | Gallego-Duran et al (36) | **Spain** | 7 centres | 51 total cohort and 50 validation cohort | 78 (62%) male total cohort and 54 (62%) validation cohort | 100% Caucasian ethnicity | 30.6 overall cohort and 31.1 validation cohort |
| 2022 | Gao et al (37) | **China** | single centre | 42.8 total cohort and 43.6 in validation | 188 (72.6%) male and 57 validation cohort (66.3%) male | n/a | 26.7 total cohort and 26.8 in validation cohort |
| 2021 | Garteiser et al (38) | **France** | single centre | 42 total and 43 NASH cohort | 128 (84) women total out of 152 with liver biopsies and NASH 19 (63) | n/a | 44.1 total and 46.6 NASH cohort |
| 2012 | Guzmán-Aroca et al (39) | **Spain** | single centre | 43.7 | 18/32 (56.3%) male | n/a | male 44.3 and female 45.1 |
| 2021 | Idilman et al (40) | **USA** | single centre | 49.2 | 42/135 (31.1%) female | n/a | 42.5 |
| 2007 | Lijima et al (41) | **JAPAN** | single centre | 59 NAFLD cohort and 45 NASH cohort | n/a | n/a | 25 NAFLD and NASH 28 |
| 2023 | Imajo et al (42) | **JAPAN + USA** | 4 centres. 2 for validation and 1 for testing and further 1 for non-Japanese cohort | 60.9 primary cohort and 61.8 validation and UCSD 52.4 | 102:74 male:female primary cohort, 23:146 M:F validation and 103:131 USCD cohort | Caucasian/Hispanic/Asian/others 0/0/176/0 primary and 0/0/176/0 validation cohort and 103/66/48/17 UCSD cohort | 28.4 Japan cohort and 27.7 validation and 31.7 USCD cohort |
| 2021 | Imajo et al (43) | **Japan** | single centre | 60 total cohort and NASH cohort 60.9 | 61% patients were male and NASH cohort 46 males | n/a | 28.8 total and 29.9 NASH |
| 2022 | Jang et al (44) | **Republic of Korea** | 5 tertiary hospitals | 38 | 69 women | n/a | 24.5 |
| 2009 | Kikuchi et al (45) | **Japan** | Single centre | 38.0 total and 39.0 NASH | 10.8% female total and 13.8% female NASH | n/a | 27.4 total and 27.6 NASH |
| 2023 | Kim NH et al (46) | **Republic of Korea** | single centre | 44.9 in steatosis group and 54.5 non-steatosis group | 34/64 non-steatosis m/f and 22/22 m/f steatosis group | n/a | 23.1  steatosis group and 26.3  non-steatosis group |
| 2022 | Kim JW at al (47) | **Republic of Korea** | Single centre | 50.9 total  NASH 53.0  Non-NASH 49.3 | 28 men and 32 women total  NASH (8 men and 17 women  s) and non-NASH (20 men and 15 women; | n/a | 29.9 total and 29.4 NASH |
| 2017 | Kim TH et al (48) | **Republic of Korea** | ^Single centre^ | NASH 36.7 and 40.0 simple steatosis | 4 : 7 NASH M: F and 10 : 5 M: F SS | n/a | NASH 29.8 and 28.7 simple steatosis |
| 2022 | Kim Y et al (49) | **Republic of Korea** | single centre | 32.8 | 13 men and 12 women | n/a | 44.6 NAFLD without NASH group only and 44.4 NASH group |
| 2020 | Lee DH et al (50) | **Republic of Korea** | single centre | 48.0 (30.0-63.0) | 43/59 m/f | n/a | 25.5 |
| 2016 | Lee HW at al (51) | **Republic of Korea** | 5 tertiary centres | 40.6 and NASH 80.0 | male 111 (60.7%) NASH 55 (58.5) | n/a | 27.9 and NASH 26.1 |
| 2022 | Lee JS at al (52) | **Republic of Korea** | 5 tertiary centres | 44 | men: 132 [52.6%] | n/a | 28.6 |
| 2022 | Lee Y-S et al (53) | **Republic of Korea** | single centre | 51 and NASH 59 | men 52 (40.9%) and NASH 25 (36.2%) | n/a | 29.7 and NASH 28.0 |
| 2020 | Lee YS et al (54) | **Republic of Korea** | Single centre | 51 | female (59.2%) | n/a | 29.7 |
| 2017 | Leporq et al (55) | **France** | single centre | 61 with biopsy‐proven NAFLD and 62 NASH | 8 women and 24 men; with biopsy‐proven NAFLD and NASH 20 ( 3 women, 17 men) | n/a | ?n/a |
| 2023 | Li J et al (56) | **USA** | single centre | 55 | 64 (62) m/f | n/a | 33 |
| 2007 | Liang et al (57) | **Taiwan** | Single centre | 30.1 total cohort and NASH 30.6 | 33 male total cohort and 30.56% male NASH | n/a | 44.6 and NASH 44.8 |
| 2021 | Lubner M et al (58) | **USA** | Single centre | 49 | 112 women and 74 men | n/a | n/a |
| 2022 | Macias J et al (59) | **Spain** | single centre | 61 and NASH 66 | 97 (67%) female and 19 (68) female | n/a | 28 and bmi 30.7 |
| 2023 | Marti-Aguado et al (60) | **Spain, Portugal and USA** | 4 testing medical centres and 2 validation centres | 56 derivation cohort and 59 validation cohort | 109 (56%) female derviation cohort and 65 (53%) validation cohrot | n/a | 29.3 derviation cohort and validation cohort 32.5 |
| 2018 | Naganawa S et al (61) | **Japan** | single centre | 64.5 | 50 men and 38 women | n/a | n/a |
| 2020 | Ozturk A et al (62) | **USA** | single centre | 50.6 total cohort and age 51.8 NASH | 54 males and NAS equal and over 5 10 male | 88 White, 2 American Indian, 6 Asian, 9 Hispanic, and 1 Black. 10 unknown for total and for NASH 17 white, 1 American Indian, 1 Asian, and 3 Hispanic and 2 unknown. | 31.4 and NASH 32.1 |
| 2017 | Park C et al (63) | **USA** | Single centre | 50.8 | 56% female total cohort | White, 48 (47.1%) Asian, 21 (20.5%) Hispanic 32 (31.4%) Other 1 (1.0%) | 30.4 |
| 2017 | Pavlides m et al (64) | **UK** | single centre | 53.4 | male 43 (60%) | n/a | 32.7 |
| 2011 | Pulzi F et al (65) | **Brazil** | Single Centre | 66 individuals (11 males), with mean (SD) of 40.7(10.9) years of age | 76.9% women NASH cohort | n/a | 46.4 total and NASH 44.6 |
| 2021 | Salvati a et al (66) | **Italy** | single centre | 46.6 total cohort, 42.5 NAFL and 47.7 NASH | 17(81%) male NAFL cohort and 53(69%) male NASH cohort | n/a | 25.5 NAFL and 28.8 NASH |
| 2021 | D'Avila da Silva a et al (67) | **Brazil** | Single centre | 39.4 total and 40.2 NASH | female total 49 (80.3) and female NASH 40 (81.6) | n/a | total 42.5±5.5 and NASH 42.1±5.7 |
| 2016 | Smits LP et al (68) | **Netherlands** | single centre | age 58 controls and 56 NASH and 50.6 steatosis | 5 men controls and 7 NASH and 10 (91) steatosis | n/a | 27.1 steatosis and 29.4 NASH |
| 2023 | Sourianarayanane A et al (69) | **USA** | single centre | 55.6 NAFLD with NASH(78) and 51.8 NAFLD without NASH (24) and 42.1 normal histology (11) | 2 (18.2%) male normal and 20 (25.6%) NAFLD without NASH and 7 (29.2%) NASH | n/a | 27.8 normal and 37.4 NAFLD without NASH and NASH 38 |
| 2002 | Saadeh S et al | **USA** | single centre | 47 NASH and steatosis 43 | 47% NASH male and 38% male steatosis | n/a | 35 NASH and 31 steatosis |
| 2020 | Sugimoto K et al | **Japan** | single centre | 53 years | 57 (52%) male | n/a | 27.2 |
| 2009 | Tarantino G et al | **Italy** | single centre | 40 NASH and 34 steatosis | 22/43 (51.2%) NASH female and 19/40 (47.5%) steatosis female | 100% caucasian | n/a |
| 2008 | Tomita K et al | **Japan** | single centre | 42 total 47 and definite NASH | 21% (4) female total and 30% (3) definite NASH | n/a | 26.5 total cohort and 27.1 definite NASH |
| 2023 | Torkzaban M et al | **USA** | Single Centre | N/a for biopsied cohort | n/a | n/a | n/a |
| 2021 | Trowell J et al | **USA** | single centre | 55.6 | 118 (54%) female | 143 (66%) white and 65 (30%) black and other 9 (3%) | 30.9 |
| 2021 | Troelstra M et al | **Amsterdam** | Single centre | 49.0 | 23 men, 14 women | n/a | 33.2 |
| 2018 | Wildman-Tobriner B et al | **USA** | 3 clinical trials | 51 | 60.1% female | 88.4% white and 29.4% hispanic | 33.3 |
| 2014 | Yilmaz Y et al | **Turkey** | single centre | 45 definite NASH and 46 other forms of NAFLD | definite NASH 73/62 M: F and other forms of NAFLD 53/47 | n/a | 31.2 definite NASH and 29.9 NAFLD |
| 2011 | Zardi em et al | **Italy** | 4 medical universities | 46.2 total and 47 NASH | 30/94 female and NASH 27/74 female | n/a | n/a |
| 2023 | Zhao y et al | **China** | single centre | 54.0 | 40/75 female | n/a | 25.8 |
| 2022 | Zhou j et al | **China** | single centre | 46 | 55 (47.4%) male | n/a | 66 (56.90) over 25 and 13 (11.20) over 30 |
| 2020 | Kim J et al | **Republic of Korea** | single centre | 51.0 total cohort and NASH 56.6 | 31 (66.0%) female and NASH 15 (75%) female | n/a | 28.3 and NASH 28.7 |
| 2016 | Loomba et al | **USA** | single centre | 50.2 | 56 (56%) female | White 46% African American 1%  Asian 17% Hispanic 32%  Multi-racial 1% and Other 1% Refused to disclose 2% | 32.1 |
| 2014 | Loomba et al | **USA** | Single centre | 50.1 | 65 (56%) female | White 52.1% Black 0.9% Asian 17.1% Hispanic 27,4%  Multi-racial 0.9% and Other 0.9% Refused to disclose 0.9% | 32.4 |
| 2015 | Parente D et al | **Brazil** | single centre | 54 | 49 (83%) female | n/a | 31.5 |
| 2020 | Nelson et al | **USA** | single centre | 47 total and NASH 52 | 58 (27.9%) female and NASH group 36 (34.3%) female | n/a | 32.9. total and NASH 33.9 |
| 2009 | Lirussi et al | **Italy** | single centre | NAFLD group 55 | NAFLD group 24 (38%) female | n/a | 30.4 |
| 2015 | Petrick et al | **USA** | single centre | 44.2 | 419 (82 %) were female | 504 (98 %) Caucasian, Hispanic 4 (1%) and Black 4 (1%) and American Indian 1 (<1 %) | 47.2 |

Abbreviations; n/a, not available; kg/m2, kilograms per metre squared; ITP

**Imaging studies and NAS ≥ 4 and F2**

Other imaging modalities that looked to diagnosis high risk NASH included ultrasound, fibroscan, CAP and MRI. The range of AUROC was from 0.60 to 0.93. The lowest was for T1 mapping by Lee Y et al. However other studies looking at T1 mapping report an AUROC of 0.74 by Imajo et al 2021 and jiahui Li et al 2023 report an AUROC of 0.72. The highest AUROC for other imaging studies and high-risk MASH was by Noureddin M et al 2022 for the MAST score which they developed from the equation 12.17 + 7.07 log MRE + 0.037 PDFF + 3.55 log AST. Imajo et al later evaluated the MAST score reporting an AUROC of 0.70 and additionally, Kim BK et al also evaluated the score reporting an AUROC of 0.72. These studies used the same cut-offs of <0.165 and ≥0.242 that were used in the original Noureddin study.

Table. Diagnostic accuracy of imaging studies in high-risk MASH diagnosis

| Other modalities | Method/model used | AUROC | Pathology | Sensitivity | Specificity |
| --- | --- | --- | --- | --- | --- |
| Sugimoto K et al 2021 s(1) | 1. (LAD NASH score) combined US markers - LS, AC, and DS  2.LS  3.DS  4.AC  5.LS+DS+AC+AST+ALT+ASTALT ratio | 1.0.88 (CI 0.80 - 0.95)  2.0.89 (CI 0.8 – 0.98)  3.0.78 (CI 0.68-0.87)  4.0.63(CI 0.50-0.76)  5.0.82(CI 0.71-0.92)  Validation cohort | FLIP NASH + NAS≥ of 4 and F ≥2 | 1.Rule out zone  01.0 and Rule in zone 0.84 | 1.Rule in zone  0.71 and Rule out zone 0.34 |
| Eilenberg et al  2021 (32) | 1. Fibroscan  2. CAP | 0.64 (CI N/a)  0.76 (CI N/a) | FLIP NASH + NAS ≥4 + F ≥2) | 1. 53.6  2. 80.3 | 1. 46.4  2. 62.1 |
| imajo et al 2023 (42) | F-CAST) (M-PAST)– and (MAST) | AUROCs of  F-CAST 0.79 (CI 0.65-0.82)  M-PAST 0.77 (CI 0.698-0.833)  MAST 0.70  (CI 0.628-0.770)  Validation cohort | NASH (FLIP/SAF) + NAS ≥4 + fibrosis stage ≥2) | N/a | N/a |
| kim BK et al 2022 s(2) | 1.MEFIB - MRIE plus Fib4 score  2.MAST | 1.0.77 (CI 0.73–0.81)  2.0.72 (CI 0.67–0.77) | at risk NASH defined as [NAS](https://www.sciencedirect.com/topics/medicine-and-dentistry/non-alcoholic-fatty-liver-disease) ≥4 and fibrosis stage ≥2  NASH was defined as scoring at least 1 in steatosis, lobular inflammation and ballooning | MAST 0.50 rule in criteria  ≥0.242  MEFIB 0.60 rule in criteria MRE ≥3.3 kPa  Mast 0.65 rule out criteria <0.165  Mefib 0.92 rule out criteria MRE <3.3 kPa | MAST 0.84 rule in criteria  ≥0.242  MEFIB 0.78 rule in criteria  0.79 mast rule out, criteria <0.165  0.56 mefib rule out MRE <3.3 kPa criteria |
| Imajo et al 2021 (43) | 1.cT1  2.MRI-PDFF  3.MRE  4.VCTE-LSM  5.CAP  6.2D-SWE  7.cT1 and MRI-PDFF  8.VCTE-LSM and CAP | 1. 0.74 (CI: 0.66-0.82)  2. 0.71 (CI: 0.63-0.80)  3.0.66 (CI: 0.57-0.75)  4.0.64 (CI: 0.54-0.74)  5.0.68 (CI: 0.59-0.78)  6.0.62 (CI: 0.49-0.75)  7.0.76 ( CI: 0.69-0.84)  8.0.70 (CI: 0.61-0.79) | NAS ≥ 4 with F ≥ 2 | N/a | N/a |
| Kuroda et al 2021 s(3) | Prediction model - logit (p) = 0.5414 × liver stiffness measurement (kPa) + 7.791 × attenuation coefficient (dB/cm/MHz)—8.401 | 0.83 (CI n/a) | NASH (FLIP) + NAS ≥4 and a fibrosis stage of ≥2 | 0.81  cutoff point of >0.30 | 0.75 |
| Lee JS et al  2022 (52) | VCTE | 0.81 (CI n/a) | NAS ≥4 + F ≥2 | 0.90  Cut off 7.7 | 0.58  Cut off 7.7 |
| Lee y et al  2020 (54) | 1.TE  2.T1 Mapping  3.MRE | 1. 0.70 (CI 0.60-0.78)  2. 0.60 (CI 0.50-0.70)  3. 0.80 (CI 0.71-0.87) | NASH was defined when >5% of hepatic steatosis and inflammation were seen with hepatocyte ballooning, regardless of the degree of fibrosis and F2 | N/a | N/a |
| Li J et al  2023 (56) | 1.MRE  2.PDFF + MRE + T1  3.PDFF + MRE  4.MRE + T1  5.T1  6. MRI-PDFF  7.PDFF + T1 | 1. 0.89 (CI: 0.82 - 0.95)  2. 0.85 (CI N/a)  3. 0.84 (CI N/a)  4. 0.81 (CI N/a)  5. 0.72 (CI N/a)  6. 0.70 (CI N/a)  7. 0.69 (CI N/a) | The histologic assessment included the presence of NASH, grade of steatosis, inflammation, ballooning, and fibrosis stage based on NASH Clinical Research Network criteria. At-risk NASH was defined as NASH with stage ≥2 fibrosis. | 1. LS was 3.3 kPa sensitivity: 0.79  2.N/a  3. N/a  4. N/a  5. cutoff value of T1 was 850 ms sensitivity 0.75  6. N/a  7. N/a | 1. cutoff value 3.3 kPa specificity 0.82  2.N/a  3.N/a  4.N/a  5. cutoff value of T1 was 850 ms specificity 0.63  6. N/a  7. N/a |
| Noureddin M et al  2022 s(4) | MRI-AST (MAST) score -12.17 + 7.07 log MRE + 0.037 PDFF + 3.55 log AST | MAST score 0.93 (CI 0.88-0.97)  Validation cohort | NAS ≥4 (with at least one point each for steatosis, lobular inflammation, and ballooning), and fibrosis stage ≥2 | cut-off of 0.242 corresponded to a sensitivity of 0.75  0.89 sensitivity at cut-off of 0.165 | cut-off of 0.242 give 0.90  cut-off of 0.165 corresponded to a specificity of 0.72 |
| Ozturk A et al 2020 (62) | 2D-SWE | 0.73 (CI 0.61–0.84) | NASH + ≥F2 (doesn’t specify a NAS score) | Cut off 8.4 kPA (1.67 m/s) with sensitivity of 0.77 | 8.4 kPA (1.67 m/s) cut- off specificity of 0.66. |
| Dennis A et al  2020 s(5) | 1.MRI cT1  2.T1, AST and Fasting glucose – cTAG risk score | 1.Ct1 0.73 (CI 0.62–0.84)  2.of 0.90 (CI 0.84–0.97) | NAS ≥4 + ≥F2 | 1. Ct1 cut off 825ms sen 0.9 and Ct1 cut off 980ms sens 0.26  2.Ctag risk score of 34% se 0.92 | 1.Ct1 cut off 825ms spec 0.40 and Ct1 cut off 980ms spe 0.9  2. Ctag risk score of 34% sp 0.79 |
| Marti-Aguado et al  2023 (60) | MR-MASH Score | 0.76 (CI 0.67–0.84) | MASH with NAS ≥4 (with at least one point each for steatosis, lobular inflammation, and ballooning), and significant fibrosis (F ≥ 2) | 0.88 | 0.56 |

FAST score

There are multiple large global studies validating the FAST score and as they contain similar definitions of MASH, they are easier to compare. The demonstrated AUROCs range from 0.69 to 0.87. The largest cohort of patients were in studies by Newsome et al with 1026 patients in the validation cohort and Chang et al with 1370 patients demonstrating AUROCs 0.85 and 0.77 respectively. Most studies used 0.35 as the rule-out cut off and 0.67 as the rule-in cut off as determined by Newsome et al who developed the model. De A et al found that different cut-offs performed better then the previously used cut-offs by Newsome et al when looking at an Indian cohort.

Table. Diagnostic accuracy of FAST score in MASH diagnosis

| Author and year | AUC (95% CL) | Path grading | Sensitivity | Specificity |
| --- | --- | --- | --- | --- |
| Anand et al 2020 s(6) | 0.79 (CI 0.74–0.84) | NASH (FLIP) + NAS ≥ 4 + F ≥ 2 | 0.90  Rule-out zone (FAST ≤ 0.35  Using the rule-out cut off of 0.35, the sensitivity of the FAST score to correctly exclude was 0.98 in the 1 year post -bariatic surgery group | 0.84  Rule-in zone (FAST ≥ 0.67) |
| Cardoso et al  2022 s(7) | 0.78 (CI 0.72–0.84) | NAS ≥ 4 + F ≥ 2 | 0.79 at 0.35  0.41 At 0.67 | 0.64 at 0.35  0.89 at 0.67 |
| Chang et al  2023 s(8) | 0.77 (CI 0.75, 0.79] | NASH (Brunt) + NAS ≥ 4 + F ≥ 2 | 0.8 [CI 0.75 - 0.85]  0.35 | 0.65 [CI 0.58 - 0.71]  0.67 |
| De A et al  2021 s(9) | 0.81 (CI: 0.68–0.90) | NASH (FLIP) + NAS ≥ 4 + F ≥ 2 | FAST: ≤ 0.35 sensitivity: 0.91  FAST: ≥ 0.67 sensitivity: 0.73  FAST: ≤ 0.55 sensitivity of 0.90  FAST: ≥ 0.78 sensitivity: 0.64, | FAST: ≤ 0.35 specificity: 0.14  FAST: ≥ 0.67 specificity: 0.67  FAST: ≤ 0.55 specificity: 0.45,  FAST: ≥ 0.78 specificity 0.94 |
| Eilenberg et al  2021 (32) | 0.76 (ITD group) (CI n/a)  0.78 (PP group) (CI n/a) | NASH (FLIP) + NAS ≥4 + F ≥2 | 1.00 (ITD group) cut off 0.206  1.00 (PP group) at cut off 0.206 | 41.5 (ITD group at cut off 0.206)  45.3 (PP group) at cut off 0.206 |
| Hirooka et al  2021 s(10) | 1.2D-SW 0.81 (CI n/a)  2. VCTE (fibroscan) 0.85 (CI n/a) | NASH (NAS) + NAS ≥4 + F ≥2 | 1.cut-off of ≤0.35 had a sensitivity of 0.91  2.a cut-off of ≤0.35 had a se of 0.92  More thresholds are available | 1.cutoff of ≥0.67 spe of 0.92  2. cutoff of ≥0.67 spe of 0.91 |
| imajo et al  2023 (43) | 0.74 (CI 0.65-0.82)  Validation cohort | NASH (FLIP/SAF) + NAS ≥4 + fibrosis stage ≥2 | N/a | N/a |
| Kim BK et al 2022 (2) | 0.69 (CI 0.64–0.73)  FAST (fibroscan) | at risk” NASH defined as [NAS](https://www.sciencedirect.com/topics/medicine-and-dentistry/non-alcoholic-fatty-liver-disease) ≥4 and fibrosis stage ≥2  NASH was defined as one in each category. | 0.32 rule in criteria ≥0.67  0.80 rule out criteria  ≤0.35 | 0.88 rule in criteria ≥0.67  0.52 rule out criteria  ≤0.35 |
| Kuroda et al 2021 (3) | 0.82 (CL N/a) | NASH (FLIP) + NAS ≥4 and a fibrosis stage of ≥2  Nash from NAFLD | N/a | N/a |
| Lee JS et al 2022 (52) | 0.71 FAST (CI, 0.65–0.78), | NASH + NAS ≥4 + F ≥2, | The cutoff values of the FAST score were obtained as 0.37 for 0.90 sensitivity  cutoff values of the FAST score (0.57), sensitivity, 69.4  (≤0.35) and rule-in zone sensitivity 0.93 (≥0.67) = | 0.79 for 0.90 specificity  cutoff values of the FAST score (0.57) spe 67.0,.  cut off (≥0.67) of the FAST score |
| Newsome P et al s(11) | 0.85 (CI 0.83 to 0.87)    pooled external validation | NASH (FLIP) + NAS ≥4 + F ≥2, | 0.89  **Rule-out zone (FAST ≤0·35)**  **0.92**  **Rule-in zone (FAST ≥0·67)** | 0.64  **Rule-out zone (FAST ≤0·35)**  **0.49**  **Rule-in zone (FAST ≥0·67)** |
| Noureddin M et al  2022 s(4) | 0.87 (0.81-0.92)  validation | NAS ≥4 (with at least one point each for steatosis, lobular inflammation, and ballooning), and fibrosis stage ≥2 | Cut off 0.35  Sen 0.66  Cut off 0.67  Sens 0.21 | Cut off 0.35  Spec 0.87  Cut off 0.67  Spec 0.99 |
| Oeda S et al  2020 s(12) s | M probe 0.76  XL probe 0.76  No auroc for validation set | FLIP NASH + NAS ≥4 + F ≥2 | M probe cut off 0.57 with 0.58 sensitivity  For the XL probe, the cut-off value was 0.56 with 0.58 sensitivity  Validation cohorts. Cut -off found using youden index  Using the cut-off value 0.35 sensitivity of FAST score with M probe was 0.90 the sensitivity was 0.86 when XL was used  In verification set. | M probe cut off 0.57  0.75  XL probe cut off 0.56  0.82  Validation cohorts  using the cut-off value 0.67 specificity with M probe was 0.92, and 0.93 when XL probe was used. |
| Tavagilone et al  2021 s(13) | 0.76 (CI 0.66–0.86) | (NASH +NAS ≥ 4 + F ≥ F2). NASH was diagnosed by Brunts criteria. | FAST ≤0.35 (rule-out zone)  Sen 0.52 (0.3 – 0.70)  fast > or equal 0.67 (rule in zone )  sen 0.09 (0-0.22) | FAST ≤0.35 (rule-out zone)  Spec 0.79 (0.71-0.88)  Fast > or equal 0.67 (rule in zone )  spec 0.98 (0.95-1) |
| woreta t et al  2022 s(14) | 0.81 (CI: 0.77 - 0.84) | NAS ≥ 4 with at least 1 point in each category and a fibrosis stage ≥ 2. | (rule-out zone) cutoff of 0.35 yielded a sensitivity of 0.91  cut off 0.67 (rule in zone) 0.51 | cut off 0.35 (rule out zone) 0.50  The rule-in zone cutoff of 0.67, specificity of 0.87, |
| Marti-Aguado et al  2023 (60) | 0.76 (CI 0.68–0.84)  Testing cohort | NAS ≥ 4 with at least 1 point in each category and a fibrosis stage ≥ 2 | N/a | N/a |

**Clinical Trials**

1. NAFLD Study: US vs Liver Biopsy; Evaluates the diagnostic accuracy of the 2DSW for the diagnosis of NAFLD and NASH in 20 obese patients (BMI> 30) candidates for laparoscopic bariatric surgery and / or cholecystectomy. The trail status is unknown. It started on September 15, 2019 with the aim for 20 participants. (15)
2. Non-invasive Rapid Assessment of NAFLD Using Magnetic Resonance Imaging With LiverMultiScan; This trial has completed recruitment with 801 patients. It started in September 2017. It is a multi-centre randomised controlled trial to determine the implementation and health care cost of LiverMultiScan vs. routine methodical assessment (standard care) of Non-alcoholic fatty liver disease (NAFLD) across several European countries. (16)
3. Non-alcoholic Fatty Liver Disease Parametric PET (FLiPP) Study; Currently active but not recruiting. The study group proposes to evaluate the role of fluorodeoxyglucose (FDG) positron emission tomography (PET) in MASH diagnosis and in combination with MRI for detection, steatosis, MASH and fibrosis in correlation to liver biopsy. (17)
4. Innovative Liver Elasticity, Attenuation, and Dispersion Ultrasound Study – This trial is currently recruiting. It started July 2019 with the aim to investigate ultrasound parameters (SW speed, Dispersion slope, Attenuation value, Normalized Local Variance, HRI) to detect fibrosis, intralobular inflammation, ballooning degeneration and steatosis and diagnostic performance of SW speed for liver fibrosis, Dispersion slope for intralobular inflammation and Attenuation value for steatosis by comparison with the tissue diagnosis by liver biopsy. Aiming for 400 participants. (18)
5. Simultaneous Quantification of Liver Fat Content, Fatty Acid Composition, and Fibrosis Using Spin-lock MRI for Steatohepatitis Assessment; This trial aims to recruit 120 subjects (60 with simple steatosis and 60 with NASH). It investigates the diagnostic value of spin-lock MRI for detecting NASH. It is not yet recruiting and its protocol was published in May 2022. (19)
6. Liver investigation: Testing marker utility in steatohepatitis (LITMUS): Assessment & validation of imaging modality performance across the NAFLD spectrum in a prospectively recruited study (the LITMUS imaging study) – This trial active. It evaluates LiverMultiScan, MRE, MRI, MRI PDFF, 2D SWE and TE will be evaluated. Its primary aim is for fibrosis diagnosis but secondary aim for MASH diagnosis and liver outcomes, steatosis and iron levels. (20)
7. Predictable MR index for Non-alcoholic Steatohepatitis (NASH) - Kim J et al 2020, already completed and published, included in our paper. (21)
8. The role of ultrasound shear wave elastography in the management of liver disease - The role of ultrasound shear wave elastography, fibroscan, and ARFI in the management of liver disease. First enrolment 03/03/2016, completed recruitment and published results but paper doesn’t involve its secondary outcome of distinguishing NASH from NAFLD. (22)

**Clinical Trial References**

1. Sugimoto K, Lee DH, Lee JY, Yu SJ, Moriyasu F, Sakamaki K, et al. Multiparametric US for Identifying Patients with High-Risk NASH: A Derivation and Validation Study. Radiology. 2021;301(3):625-34.

2. Kim BK, Tamaki N, Imajo K, Yoneda M, Sutter N, Jung J, et al. Head-to-head comparison between MEFIB, MAST, and FAST for detecting stage 2 fibrosis or higher among patients with NAFLD. J Hepatol. 2022;77(6):1482-90.

3. Kuroda H, Fujiwara Y, Abe T, Nagasawa T, Oguri T, Noguchi S, et al. Two-dimensional shear wave elastography and ultrasound-guided attenuation parameter for progressive non-alcoholic steatohepatitis. PLoS One. 2021;16(4):e0249493.

4. Noureddin M, Truong E, Gornbein JA, Saouaf R, Guindi M, Todo T, et al. MRI-based (MAST) score accurately identifies patients with NASH and significant fibrosis. J Hepatol. 2022;76(4):781-7.

5. Dennis A, Mouchti S, Kelly M, Fallowfield JA, Hirschfield G, Pavlides M, et al. A composite biomarker using multiparametric magnetic resonance imaging and blood analytes accurately identifies patients with non-alcoholic steatohepatitis and significant fibrosis. Sci Rep. 2020;10(1):15308.

6. Anand A, Elhence A, Vaishnav M, Singh AA, Rajput MS, Banyal V, et al. FibroScan-aspartate aminotransferase score in an Asian cohort of non-alcoholic fatty liver disease and its utility in predicting histological resolution with bariatric surgery. J Gastroenterol Hepatol. 2021;36(5):1309-16.

7. Cardoso AC, Tovo CV, Leite NC, El Bacha IA, Calcado FL, Coral GP, et al. Validation and Performance of FibroScan-AST (FAST) Score on a Brazilian Population with Nonalcoholic Fatty Liver Disease. Digestive Diseases and Sciences. 2022;67(11):5272-9.

8. Chang D, Truong E, Mena EA, Pacheco F, Wong M, Guindi M, et al. Machine learning models are superior to noninvasive tests in identifying clinically significant stages of NAFLD and NAFLD-related cirrhosis. Hepatology. 2023;77(2):546-57.

9. De A, Mishra S, Keisham A, Mehta M, Verma N, Premkumar M, et al. Fibroscan-ast (FAST) score for non-alcoholic steatohepatitis (NASH)-validation in an Indian cohort. Hepatology. 2020;72(1 SUPPL):916A.

10. Hirooka M, Koizumi Y, Yano R, Sunago K, Watanabe T, Yoshida O, et al. Validation of the FibroScan-aspartate aminotransferase score by vibration-controlled transient and B-mode ultrasound elastography. Hepatol Res. 2021;51(6):652-61.

11. Newsome PN, Sasso M, Deeks JJ, Paredes A, Boursier J, Chan WK, et al. FibroScan-AST (FAST) score for the non-invasive identification of patients with non-alcoholic steatohepatitis with significant activity and fibrosis: a prospective derivation and global validation study. Lancet Gastroenterol Hepatol. 2020;5(4):362-73.

12. Oeda S, Takahashi H, Imajo K, Seko Y, Kobayashi T, Ogawa Y, et al. Diagnostic accuracy of FibroScan-AST score to identify non-alcoholic steatohepatitis with significant activity and fibrosis in Japanese patients with non-alcoholic fatty liver disease: Comparison between M and XL probes. Hepatol Res. 2020;50(7):831-9.

13. Tavaglione F, De Vincentis A, Bruni V, Gallo IF, Carotti S, Tuccinardi D, et al. Accuracy of controlled attenuation parameter for assessing liver steatosis in individuals with morbid obesity before bariatric surgery. Liver Int. 2022;42(2):374-83.

14. Woreta TA, Van Natta ML, Lazo M, Krishnan A, Neuschwander-Tetri BA, Loomba R, et al. Validation of the accuracy of the FAST™ score for detecting patients with at-risk nonalcoholic steatohepatitis (NASH) in a North American cohort and comparison to other non-invasive algorithms. PLoS One. 2022;17(4):e0266859.

15. Nct. NAFLD Study: US vs Liver Biopsy. <https://clinicaltrialsgov/show/NCT04101162>. 2019.

16. Dollinger M. Non-invasive Rapid Assessment of NAFLD Using Magnetic Resonance Imaging With LiverMultiScan (RADIcAL1) [Available from: <https://clinicaltrials.gov/study/NCT03289897>.

17. University of California D, Diabetes NIo, Digestive, Diseases K. Non-alcoholic Fatty Liver Disease Parametric PET (FLiPP) Study. <https://classic.clinicaltrials.gov/show/NCT02754037>; 2016.

18. University TM. Innovative Liver Elasticity, Attenuation, and Dispersion Ultrasound Study. <https://classic.clinicaltrials.gov/show/NCT04012242>; 2019.

19. Kong CUoH. Simultaneous Quantification of Liver Fat Content, Fatty Acid Composition, and Fibrosis Using Spin-lock MRI for Steatohepatitis Assessment. <https://classic.clinicaltrials.gov/show/NCT05384652>; 2024.

20. Pavlides M, Mozes FE, Akhtar S, Wonders K, Cobbold J, Tunnicliffe EM, et al. Liver investigation: Testing marker utility in steatohepatitis (LITMUS): Assessment & validation of imaging modality performance across the NAFLD spectrum in a prospectively recruited cohort study (the LITMUS imaging study): Study protocol. Contemporary Clinical Trials. 2023;134 (no pagination).

21. Kim JW, Lee YS, Park YS, Kim BH, Lee SY, Yeon JE, et al. Multiparametric MR Index for the Diagnosis of Non-Alcoholic Steatohepatitis in Patients with Non-Alcoholic Fatty Liver Disease. Sci Rep. 2020;10(1):2671.

22. Atzori SM, Pasha Y, Maurice JB, Taylor-Robinson SD, Campbell L, Lim AKP. Prospective evaluation of liver shearwave elastography measurements with 3 different technologies and same day liver biopsy in patients with chronic liver disease. Dig Liver Dis. 2024;56(3):484-94.
